# CBL0137 and NKG2A blockade: a novel immuno-oncology combination therapy for Myc-overexpressing triple-negative breast cancers

**DOI:** 10.1038/s41388-024-03259-y

**Published:** 2024-12-21

**Authors:** Prahlad V. Raninga, Bijun Zeng, Davide Moi, Ethan Trethowan, Federica Saletta, Pooja Venkat, Chelsea Mayoh, Rochelle C. J. D’Souza, Bryan W. Day, Tyler Shai-Hee, Orazio Vittorio, Roberta Mazzieri, Riccardo Dolcetti, Kum Kum Khanna

**Affiliations:** 1 QIMR Berghofer Medical Research Institute, 300 Herston Road, Herston, Brisbane, QLD 4006 Australia; 2 Mater Research Institute, The University of Queensland, Translational Research Institute, Woolloongabba, QLD 4102 Australia; 3 Peter McCallum Cancer Centre, 305 Grattan Street, Melbourne, VIC 3000 Australia; 4 Sir Peter MacCallum Department of Oncology, The University of Melbourne, Melbourne, VIC Australia; 5 Children’s Cancer Institute, Lowy Cancer Research Centre, UNSW, Kensington, NSW Australia; 6 Murdoch Children’s Research Institute, 50 Flemington Road, Parkville, VIC Australia; 7 School of Clinical Medicine, UNSW Medicine & Health, UNSW Sydney, Kensington, NSW Australia; 8 Department of Microbiology and Immunology, The University of Melbourne, Melbourne, VIC Australia

**Keywords:** Breast cancer, Targeted therapies

## Abstract

The *MYC* proto-oncogene is upregulated in >60% of triple-negative breast cancers (TNBCs), it can directly promote tumor cell proliferation, and its overexpression negatively regulates anti-tumor immune responses. For all these reasons, *MYC* has long been considered as a compelling therapeutic target. However, pharmacological inhibition of MYC function has proven difficult due to a lack of a drug-binding pocket. Here, we demonstrate that the potent abrogation of MYC gene transcription by CBL0137 induces immunogenic cell death and reduces proliferation in MYC-high but not in MYC-low TNBC in vitro. CBL0137 also significantly inhibited the in vivo growth of primary tumors in a human MYC-high TNBC xenograft model (MDA-MB-231). Moreover, CBL0137 inhibited the tumor growth of highly aggressive mouse 4T1.2 syngeneic TNBC model in immunocompetent mice by inhibiting the MYC pathway and inducing Type I interferon responses. Immune profiling of CBL0137-treated mice revealed significantly enhanced tumor-specific immune responses and increased proportions of tumor infiltrating effector CD8^+^ T cells, CD4^+^ T cells, and NK cells. CBL0137-induced immune activation also resulted in increased exhaustion of immune effector cells. In particular, NKG2A up-regulation on activated effector cells and of its ligand Qa-1^b^ on tumors in vivo was identified as a possible immune evasive mechanism. Indeed, NKG2A blockade synergized with CBL0137 significantly inhibiting the in vivo growth of 4T1.2 tumors. Collectively, our findings provide the rationale supporting the exploitation of CBL0137-induced anti-tumor immunity in combination with NKG2A blockade to improve the treatment of TNBC expressing high levels of MYC.

## Introduction

Metastatic breast cancer (BC) still has a poor prognosis, with 3-year survival rates of just 30–40%. Among the different BC subtypes, Triple Negative Breast Cancer (TNBC) continues to have the highest rates of recurrence and mortality. This subtype tends to affect younger women, is not suitable for molecular-targeted therapy and frequently relapses after chemotherapy, with a tendency for hematogenous spread to the brain, liver, lungs, and bones [1]. New and more effective therapeutic strategies are therefore needed to improve the control of these aggressive tumors.

MYC is an oncogenic transcription factor that regulates the expression of multiple genes involved in cell proliferation, apoptosis, and metabolism [2–5]. MYC gene is amplified in several human cancers and is often associated with aggressive tumor growth, increased metastases, and chemoresistance [6, 7]. The MYC oncogenic pathway is activated in ~50–55% of breast cancers (BCs) with ~62% of TNBCs showing MYC oncogene amplification [8], suggesting that MYC represents an attractive, although difficult therapeutic target for TNBC. Several efforts have been made to target MYC using small molecule inhibitors, however none have progressed into clinical trials [9–11]. These molecules either bind directly to MYC protein or interfere with MYC/MAX dimerization. An alternative therapeutic strategy is the exploitation of synthetic lethality through the identification of the genes required for MYC oncogenic activity and their selective targeting with small molecule inhibitors. Studies have shown that targeting PIM1 kinase and BRD4 using specific inhibitors exerted synthetic lethality in MYC-amplified TNBC [10, 12]. BRD4 bromodomain inhibitors including JQ1 and I-BET-726 have gained particular interest and have been shown to transcriptionally inhibit MYC and exert therapeutic activity against several cancers [9, 13]. Therefore, drugs that inhibit MYC-dependent transcription may be of therapeutic relevance in controlling MYC-overexpressing and highly aggressive tumors such as TNBCs.

In this respect, CBL0137, a non-genotoxic anti-cancer small molecule belonging to the curaxins family of drugs [14, 15] may be a particularly attractive drug to improve the treatment of MYC-overexpressing TNBCs. CBL0137 exerts its anti-cancer activity by intercalating into DNA and interfering with DNA-histone interactions. This induces genome-wide unfolding of nucleosomes, and the resulting chromatin-trapping of histone chaperone Facilitates Chromatin Transcription (FACT) complex in particular SSRP1 which alters global cellular transcriptional and replication programs. This results in the inhibition of key cell survival signaling pathways leading to cell death [15–18]. SSRP1 is often upregulated in multiple cancers and has a well characterized role in assisting RNA Pol II during transcription elongation. SSRP1 is known to bind and regulate promoters of multiple oncogenes including MYC [17]. In neuroblastoma, SSRP1 transcriptionally regulates N-MYC expression and pharmacological inhibition of SSRP1 using CBL0137 retarded neuroblastoma growth via N-MYC inhibition [17, 19]. Moreover, CBL0137-induced chromatin damage increases heterochromatin transcription that stimulates double-stranded RNA-induced interferon responses in tumors [20]. Additionally, CBL0137 has been shown to affect the spatial genome organization and inhibit enhancer-promoter communication, which resulted in transcriptional inhibition of MYC and many other key oncogenic transcription factors [21, 22]. Therefore, CBL0137 may lead to MYC transcriptional inhibition also in MYC-overexpressing TNBCs, possibly resulting in their growth inhibition.

This hypothesis became particularly attractive when considering emerging evidence correlating MYC overexpression with the generation of an immune suppressed tumor microenvironment [4, 23]. Indeed, MYC upregulates mRNA expression of inhibitory immune checkpoints including CD47 and PD-L1 on tumor cells [24]. MYC also negatively regulates anti-tumor immunity by favoring macrophage influx and decreasing the intratumor infiltration of CD3^+^ T cells, B220^+^ B cells, and NKp46^+^ natural killer (NK) cells [25]. Moreover, aberrant MYC expression significantly downregulates MHC Class I molecules thereby reducing the ability of tumor cells to present antigens and be recognized by T lymphocytes [26, 27]. In addition, in TNBC, MYC was shown to promote an immune-suppressive microenvironment by inhibiting the interferon gamma (IFNγ) pathway [8]. On these grounds, there is a strong rationale to investigate whether a drug such as CBL0137 is also able to counteract MYC-mediated immune suppression and promote anti-tumor immunity. Clinical trials of CBL0137 alone or in various combinations are ongoing for some cancers including CNS tumors, melanoma, sarcoma and lymphoma. Therefore, characterization of the immune-modulatory effects induced by CBL0137 in preclinical models of TNBC might provide important insights into how best to combine CBL0137 with other agents.

In this study, we show that CBL0137 induces apoptotic responses and immunogenic cell death (ICD) in MYC-high, but not in MYC-low, TNBC cells via transcriptional downregulation of MYC and its target genes. In immunocompromised mice injected with the human MYC-high MDA-MB-231 TNBC cells, CBL0137 directly and significantly inhibited the growth of primary tumors. Moreover, in the immunocompetent murine 4T1.2 TNBC model, CBL0137 inhibited the primary tumor growth by inhibiting the MYC signaling pathway together with the induction of Type I interferon responses. In this immunocompetent model, CBL0137 enhanced intratumor infiltration of activated CD8^+^ T cells and NK cells. However, CBL0137 also induced upregulation of inhibitory immune checkpoint molecules, including NKG2A and TIGIT on immune effector cells. Remarkably, combined treatment with CBL0137 and anti-NKG2A antibody resulted in synergistic tumor growth inhibition in the highly aggressive 4T1.2 TNBC model. Overall, these findings support the exploitation of CBL0137-mediated MYC downregulation and consequent induction of anti-tumor immunity to improve the treatment of TNBC expressing high levels of MYC. The rational selection of the target immune checkpoint molecules, such as NKG2A, may allow the design of more effective combination immunotherapies for these patients.

## Results

### CBL0137 inhibits proliferation in MYC-high triple-negative breast cancer cells

**Fig. 1:**
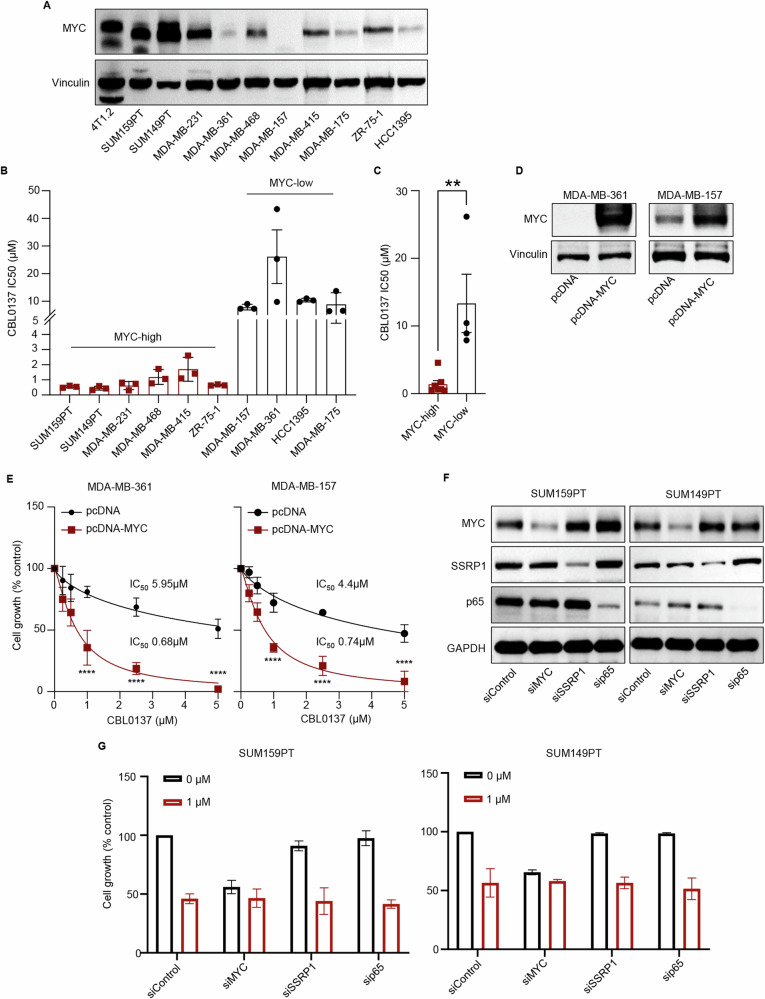
CBL0137 inhibits proliferation in MYC-high triple-negative breast cancer cells. **A** MYC protein levels were analyzed in a panel of breast cancer cell lines using Western blot. Actin was used as a loading control. **B** A panel of breast cancer cell lines were treated with CBL0137 (0–5 µM) for 72 h. Cell viability was assessed by MT cell viability assays and the IC50 value of CBL0137 in each MYC-high and MYC-low breast cancer cell line is shown. Data are presented as mean ± SEM (*n* = 3 technical replicates). **C** Mean IC50 value of CBL0137 in MYC-high and MYC-low breast cancer cell lines. Data are presented as mean ± SEM; *t* Test, **p < 0.01. **D**, **E** MDA-MB-361 and MDA-MB-157 cells were transfected with either pcDNA4 vector or pcDNA4-MYC plasmid for 24 h. MYC protein levels were analyzed by Western blot (**D**). Transfected cells were then treated with CBL0137 (0–5 µM) for 72 h. Cell viability was analyzed by MT cell viability assay (**E**) (*n* = 3 technical replicates). Data are presented as mean ± SEM; Two-Way ANOVA with Sidak’s multiple comparison test, ****p < 0.0001. **F** SUM159PT and SUM149PT cells were transfected with either scramble or control, MYC-specific, SSRP1-specific, or p65-specific siRNA for 48 h. MYC, SSRP1, and p65 protein levels were analyzed by Western blot. **G** SUM159PT and SUM149PT cells transfected with either scramble or control, MYC-specific, SSRP1-specific, or p65-specific siRNA for 24 h followed by the treatment with or without CBL0137 (1 µM) for 96 h. Cell proliferation was analysed by the MTS assays (*n* = 2 biological replicates). Data are presented as mean ± SD.

Considering that MYC mediates tumor intrinsic malignant properties, as a first step, we aimed at characterizing the biologic effects of CBL0137 in TNBC cells. Analysis of a panel of 10 TNBC cell lines (Fig. 1A) showed that CBL0137 significantly inhibited the proliferation of MYC-high (4/6 TNBC) compared to MYC-low (2/4 TNBC) cell lines (Fig. S1A). MYC-high TNBC cell lines were significantly more sensitive to CBL0137 (Mean IC50 1.39 µM ± SEM 0.56) than MYC-low TNBC cells (Mean IC50 13.35 µM ± SEM 4.3) (Fig. 1B, C). Moreover, CBL0137 significantly enhanced death of MYC-high cells (Mean Fold Increase 4.15 ± SEM 0.53 at 1 µM CBL) when compared with MYC-low cells (Mean Fold Increase 1.272 ± SEM 0.18 at 1 µM CBL) (Fig. S1B, S1C).

To investigate whether CBL0137 selectively inhibits the growth of MYC-high TNBC cells, MYC was ectopically over-expressed in two MYC-low cell lines, MDA-MB-361 and MDA-MB-157 (Fig. 1D) that were then treated with different concentrations of CBL0137 for 72 h. MYC over-expression itself did not affect cell survival, however, it significantly sensitized both MDA-MB-361 and MDA-MB-157 cells to CBL0137-mediated growth inhibition as compared to parental cells (Fig. 1E). In addition to the transcriptional downregulation of MYC [22], CBL0137 is known to exert anti-cancer activity by inhibiting the NF-kβ pathway and via trapping of SSRP1 into chromatin [15, 17, 18]. Hence, to verify in the TNBC cell context whether CBL0137 effect is dependent on MYC, NF-kβ or SSRP1, either MYC, SSRP1, or p65 gene expression was down-regulated by siRNA in MYC-high SUM159PT and SUM149PT cells (Fig. 1F). Interestingly, siRNA-mediated MYC knockdown reduced cell growth by approximately 50–60% in these two MYC-high TNBC (SUM159PT and SUM149PT) cell lines which was comparable to CBL0137 (1 µM) treatment alone in cells transfected with scramble/control siRNAs (Fig. 1G), suggesting that MYC-high lines have survival-dependency on MYC. Interestingly, CBL0137 treatment did not further reduced cell growth in SUM159PT and SUM149PT cells after MYC knockdown, suggesting that CBL0137 effect is epistatic with MYC-depletion in MYC-high TNBC lines (Fig. 1G). In contrast, SSRP1 and p65 knockdown had no effect on cell growth in both MYC-high TNBC cell lines and CBL0137 treatment exerted a comparable growth inhibitory effect in control and SSRP1 or p65 knocked down (siRNA) SUM159PT and SUM149PT cells (Fig. 1G). Taken together, these data suggested that in TNBC cell context, CBL0137-mediated growth inhibition is MYC dependent and SSRP1 and p65 independent. These results are consistent with the survival dependency of MYC-high TNBC cell lines on MYC but not SSRP1 and p65 (Fig. S1D). Lack of NF-kβ and SSRP1 involvement was further demonstrated at the protein level in SUM159PT and SUM149PT TNBC cells treated with 2.5 µM CBL0137 for 0, 4, and 8 h. Although CBL0137 markedly reduced MYC protein levels within 4 h of treatment in both cell lines (Fig. S1E), the drug did not change SSRP1 protein levels in the whole cell lysates and did not inhibit phospho-p65 levels in either cell line (Fig. S1E). Since CBL0137 is known to trap SSRP1 into the chromatin and block RNA Pol II-mediated transcription [15–18], SSRP1 and MYC protein levels were also analyzed in the chromatin fraction of SUM159PT cells. Unlike previous studies, CBL0137 did not trap SSRP1 into the chromatin within 4 h of treatment but significantly reduced the levels of MYC protein in the chromatin fraction (Fig. S1F). These data are consistent with more recent studies showing that CBL0137 can transcriptionally downregulate MYC expression via inhibition of the enhancer-promoter communication in neuroblastoma and medulloblastoma [17, 19, 22]. Together these data indicate that CBL0137 inhibits the growth of MYC-high TNBC cells in a MYC-dependent, but NF-kβ- and SSRP1-independent manner.

Next, we investigated whether MYC down-regulation was associated with the modulation of known MYC target proteins in CBL0137-treated SUM159PT cells. Eight hours of CBL0137 treatment markedly reduced the protein levels of known MYC targets including cell cycle regulating proteins CDK2, Cyclin A, and Cyclin B1 in SUM159PT cells (Fig. S1G). Moreover, four-hour CBL0137 treatment significantly reduced MYC mRNA levels by 90% compared to untreated cells in both SUM159PT (Fig. S1H) and SUM149PT (Fig. S1I) cells. Additionally, CBL0137 also significantly reduced the mRNA levels of several MYC target genes in both cell lines, including MAT2A, PP1A, HK2, PGK1, RPL5, RPL9, PYCR1, and CDK2 (Fig. S1H, S1I). These findings indicate that CBL0137 inhibits proliferation of human MYC-high TNBC cells by strongly down-regulating MYC and its downstream targets at the transcriptional level.

### CBL0137 inhibits TNBC growth in vivo *via* both direct cytotoxicity and immune modulation

**Fig. 2:**
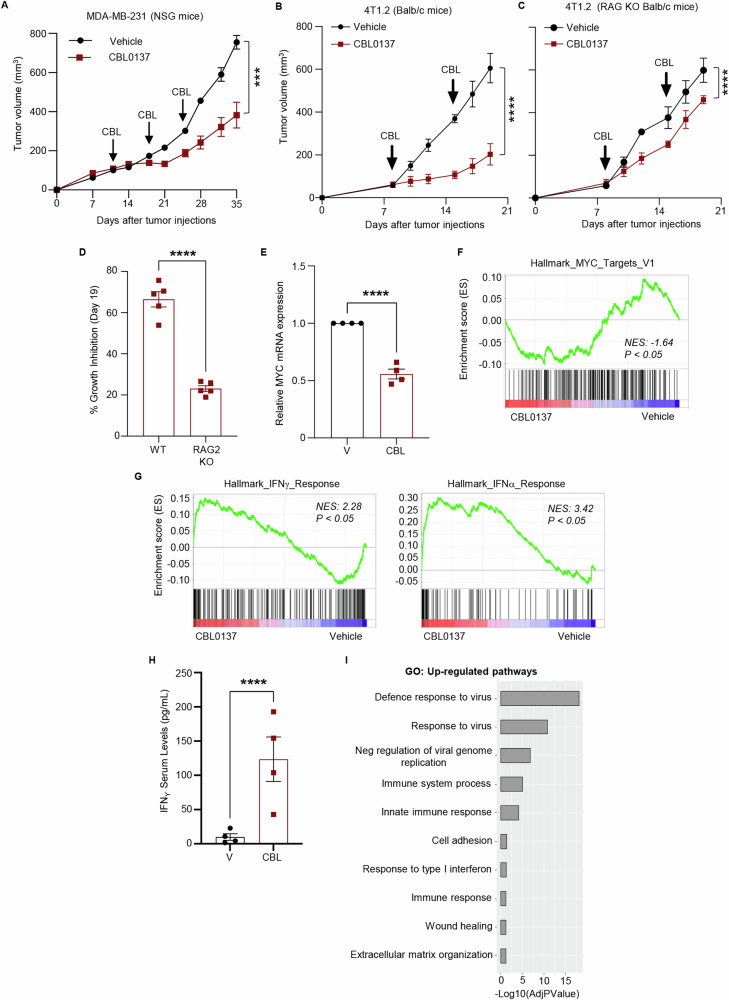
CBL0137 inhibits tumor growth, downregulates MYC, and induces inflammatory response in the in vivo breast models. **A** Tumor growth in NSG mice orthotopically injected with MDA-MB-231 cells following treatment with vehicle or CBL0137 (60 mg/kg, once/week, i.v.) for three weeks. Treatment started when the tumor reached 50-100 mm^3^. Data are presented as mean ± SEM (*n* = 6 mice/group); *t* Test on tumor volumes at day 35, ***p < 0.001. Murine 4T1.2 tumor growth in fully immunocompetent Balb/c mice (**B**) and in RAG2 knockout Balb/c mice (**C**) following treatment with vehicle or CBL0137 (60 mg/kg, once/week, i.v.) for three weeks. Data are presented as mean ± SEM (*n* = 6 mice/group); *t* Test on tumor volumes at day 21, ****p < 0.0001. **D** % Growth inhibition of murine 4T1.2 tumors in fully immunocompetent Balb/c mice (WT) and in RAG2 knockout (RAG2 KO) mice following treatment with CBL0137 for 19 days. Data are presented as mean ± SEM (*n* = 5 tumor/group); *t* Test, ****p < 0.0001. **E** Myc mRNA levels in murine 4T1.2 tumors treated with vehicle or CBL0137 were analyzed by RT-qPCR. Data are presented as mean ± SEM (*n* = 4 tumor/group); *t* Test, ****p < 0.0001. **F** Murine 4T1.2 tumor-bearing Balb/c mice were treated with vehicle or CBL0137 (60 mg/kg, IV) for one week and tumors were collected for RNA seq analysis (*n* = 4 tumor/group). Gene Set Enrichment Analysis showing downregulation of the MYC target V1 gene signature. **G** Gene Set Enrichment Analysis of the RNA Seq in murine 4T1.2 tumors treated with vehicle or CBL0137 showing upregulation of the Hallmark IFNγ and the Hallmark IFNα gene signature. **H** IFNγ levels measured by ELISA in the serum of 4T1.2 tumor-bearing mice following one week of treatment with vehicle or CBL0137 (60 mg/kg, i.v.). Data are presented as mean ± SEM (*n* = 4 mice/group); *t* Test, *p < 0.05. **I** Gene Ontology analysis (RNA-seq) of the pathways upregulated in CBL0137-treated 4T1.2 tumors compared to vehicle-treated tumors in vivo (*n* = 4 tumor/group).

We next analyzed the direct in vivo anti-cancer activity of CBL0137 using the MYC-high MDA-MB-231 TNBC cells (Fig. 1A) orthotopically injected into immunocompromised NSG mice [28, 29]. CBL0137 significantly reduced primary tumor growth (Fig. 2A, S2A) when compared to the vehicle-treated group, with no evident signs of systemic toxicity (Fig. S2B). Therapeutic efficacy of CBL0137 was also tested in the fully immunocompetent and syngeneic 4T1.2 mouse model of MYC-high TNBC (Fig. 1A) that aptly recapitulates human TNBC features including spontaneous metastasis upon orthotopic injection [30]. In vitro data confirmed the anti-proliferative effects of CBL0137 on 4T1.2 cells (IC50 0.74 µM) (Fig. S2C). In vivo, CBL0137 significantly reduced 4T1.2 tumor growth when compared to vehicle-treated mice (Fig. 2B). Since CBL0137 has been recently shown to exert part of its anti-tumor activity by increasing CD8^+^ T-cell intratumor infiltration and activation in a murine model of colon carcinoma [31], we investigated whether CBL0137 anti-tumor activity in the 4T1.2 model is also at least partly dependent on modulation of adaptive immunity. Towards this goal, 4T1.2 cells were orthotopically injected into Balb/c RAG2 knockout (KO) or WT mice (28) and treated with CBL0137. Although CBL0137 significantly reduced 4T1.2 tumor growth in both mouse strains (Fig. 2B, C), the extent of growth inhibition was significantly higher in fully immunocompetent mice (67% vs 25% at day 18) (Fig. 2D). These data suggest that the anti-tumor effects of CBL0137 in the 4T1.2 model are at least in part mediated by T cells. The observation that no change was observed in the intra-tumoral infiltration of B cells in CBL0137-treated mice (see below and Fig. S8E, F) does not support a contribution of B lymphocytes to the therapeutic efficacy of the drug.

To decipher the mechanism mediating the in vivo anti-cancer activity of CBL0137, we first confirmed down-regulation of *Myc* mRNA in vivo by RT-qPCR in 4T1.2 tumors collected 7 days after a single CBL0137 treatment (Fig. 2E). Bulk RNA sequencing (RNA-seq) and Gene Set Enrichment Analysis (GSEA) also confirmed down-regulation of MYC-target genes in the same tumors as compared with controls (*n* = 4/group) (Fig. 2F; Fig. S3A; Supplementary Data File 1). Interestingly, a significant upregulation of genes associated with both IFNγ- and IFNα-induced responses was also observed in CBL0137-treated tumors (Fig. 2G). Moreover, a single dose of CBL0137 significantly increased IFNγ levels in the serum of 4T1.2 tumor-bearing mice (Fig. 2H). The upregulation of key IFNγ pathway genes (*Oas2, Oas3, Ifit1, Ifit2, Ifit3, Ifih1, and Irf7*) in CBL0137-treated 4T1.2 tumors was independently validated by RT-qPCR (Fig. S3B). CBL0137-mediated modulation of immune-related genes was further highlighted by Gene Ontology (GO) pathway analysis, where 7 out of 10 most upregulated pathways were associated with immune responses, including responses to viruses and Type I interferon (Fig. 2I). Moreover, mRNAs encoding for cytokines (*Il-1b*), chemokines (*Ccr2* and *Cxcl9*) as well as cell surface receptors involved in innate immunity (*Clec7a* and *Clec12a*) were among those most significantly downregulated in CBL0137-treated 4T1.2 tumors (Fig. S3A, S3C). Downregulation of these genes has been shown to activate anti-tumor immune responses in multiple cancer models [32–34]. These data demonstrated the ability of CBL0137 to induce a robust interferon-mediated response and immune-stimulating effects in a murine model of MYC-high TNBC cells in vivo.

### CBL0137 induces immunogenic cell death in human and murine breast cancer cell lines

**Fig. 3:**
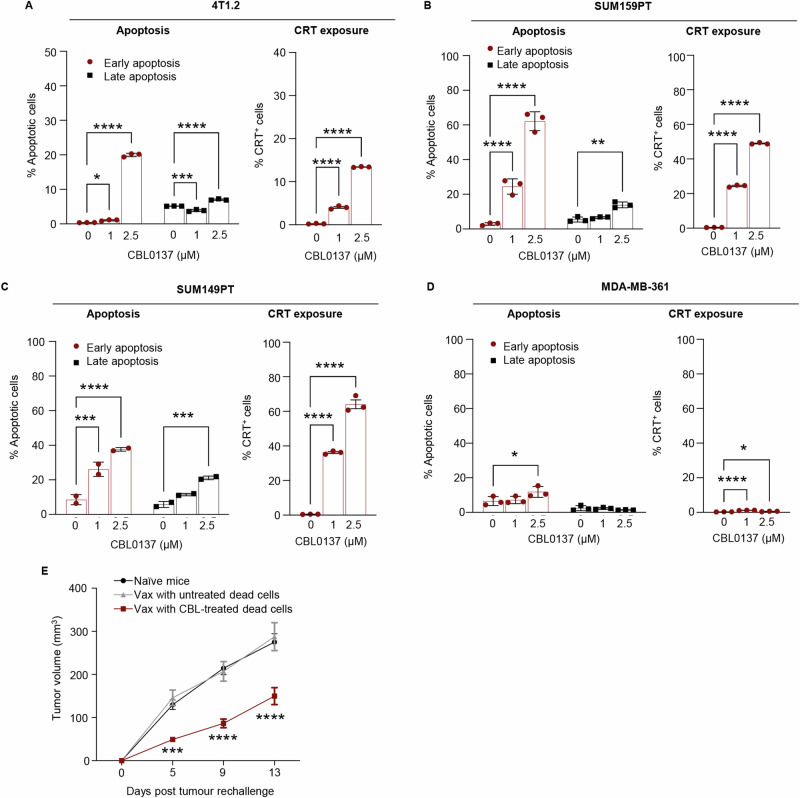
CBL0137 treatment induces immunogenic cell death in MYC-high breast cancer cells in vitro and in vivo. Murine 4T1.2 cells (**A**), human MYC-high breast cancer cells SUM159PT (**B**) and SUM149PT (**C**) as well as MYC-low MDA-MB-361 (**D**) cells were treated with CBL0137 (0–2.5 µM) for 24 h. The percentage of early apoptotic (Annexin V^+^7-AAD^-^) and late apoptotic (Annexin V^+^7-AAD^+^) cells (left) and the percentage of Calreticulin-positive (CRT^+^) early apoptotic cells (right) were analyzed by Flow Cytometry as described in methods. Data are presented as mean ± SEM (*n* = 3 technical replicates); One-way ANOVA with Dunnett’s multiple comparisons test, *p < 0.05, ***p < 0.001****p < 0.0001. **E** 4T1.2 cells killed by freezing and thawing cycles (red) or treated with 5 µM CBL0137 for 24 h (blue) were injected into the 4th mammary fat-pad of female Balb/c mice. Four days later, mice were re-challenged with untreated live 4T1.2 cells by injection into the 9th mammary fat-pad and mice were monitored for tumor growth. Naïve mice were used as negative controls (black). Data are presented as mean ± SEM (*n* = 6 mice/group); *t* Test on day 5, 9, and 13 versus Naïve mice, ***p < 0.001****p < 0.0001.

The in vivo immunostimulatory effects and the induction of type I IFN responses described above prompted us to investigate whether CBL0137 triggers immunogenic cell death (ICD) in TNBC cells. CBL0137 induced a significant and dose-dependent increase in the proportion of 4T1.2 apoptotic cells, particularly the percentage of early apoptotic cells expressing Calreticulin on their cell surface (Figs. 3A, S4A, S5A, S5B) a known ICD marker [35]. Of note, CBL0137 also induced ICD markers in human TNBC cell lines expressing high levels of MYC (Figs. 3B, 3C, S4B, S5C, S5D), with markedly reduced effects in a MYC-low cell lines (Fig. 3D). Moreover, we also examined the effect of CBL0137 treatment on HMGB1 expression, a late marker of ICD [36], on MYC-high SUM159PT and SUM149PT TNBC cells. CBL0137 treatment significantly increased HMGB1 release in SUM159PT and SUM149PT cells in a concentration-dependent manner (Fig. S6A). To further confirm if CBL0137 induces ICD via MYC downregulation, we examined if siRNA-induced MYC downregulation induces ICD in MYC-high TNBC cells. To this end, SUM159PT and SUM149PT cells were transfected with either control or MYC-specific siRNAs, and HMGB1 release was analyzed 48 h post-transfection. Results showed that MYC downregulation significantly increased HMGB1 release in both SUM159PT and SUM149PT TNBC cells, suggesting that MYC downregulation induces ICD in MYC-high TNBC cells (Fig. S6B). These data strongly support CBL0137-mediated induction of ICD via MYC downregulation in both murine and human TNBC cells expressing abnormally high levels of MYC. To characterize the immunogenicity of CBL0137-treated cells, we investigated their ability to elicit an anti-tumor immune response in vivo to protect mice from re-challenge with live tumor cells. As shown in Fig. 3E, mice injected with CBL0137-treated 4T1.2 TNBC apoptotic cells showed a significant delay in the growth of tumors in re-challenged mice when compared with those previously injected with 4T1.2 cells killed by freezing and thawing or naïve mice. These data together suggest that CBL0137 induces ICD which stimulates a partially protective anti-tumor immunity in vivo.

### In vivo CBL0137 treatment induces tumor-specific immune responses and local immunomodulation

**Fig. 4:**
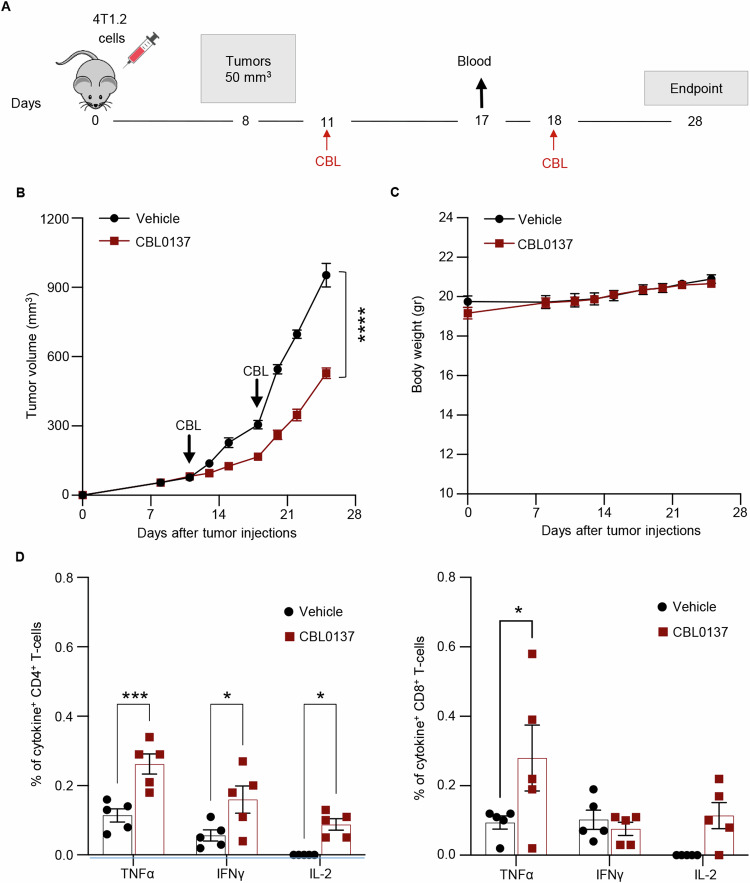
CBL0137 treatment induces tumor-specific immune response in 4T1.2 model in vivo. **A** Experimental scheme: Balb/c mice were orthotopically injected with 4T1.2 cells and 2 weekly treatments with CBL0137 (60 mg/kg) were started after the tumors reached a size of at least 50 mm^3^. Control mice received vehicle only. Blood was collected at day 17 for the polyfunctional assay and tumors were collected at day 28 for immune profiling by flow cytometry (Fig. 6). Tumor growth over time measured by caliper (**B**) and body weight (**C**) were monitored for 28 days. Data are presented as mean ± SEM (*n* = 6 mice/group); *t* Test on day 28 versus control mice, ****p < 0.0001. **D** Percentage of CD4^+^ (left) and CD8^+^ (right) T cells expressing TNFα, IFNγ and IL2 upon 6 h of ex-vivo restimulation with peptides derived from universal tumor antigens: **mSurvivin53–67** DLAQCFFCFKELEGW; **mSurvivin 66–74** GWEPDDNPI; **mTERT 167–175** AYQVCGSPL. Data are presented as mean ± SEM (*n* = 5 mice/group); One-way ANOVA with Dunnett’s multiple comparisons test, *p < 0.05, ***p < 0.001.

To investigate the ability of CBL0137 to stimulate the generation of tumor-specific T cell responses in vivo, Balb/c mice were orthotopically injected with 4T1.2 TNBC cells and treated as described before with 2 weekly injections of CBL0137 (Fig. 4A). After confirming tumor growth inhibition in the absence of evident toxicity (Fig. 4B, C), spleens were collected from treated and control mice, and splenocytes were re-stimulated ex vivo with CD4 and CD8 epitope peptides derived from universal tumor associated antigens (Telomerase and Survivin). Flow cytometry analysis on re-stimulated splenocytes, showed that mice treated with CBL0137 had significantly higher numbers of tumor-specific T cells, as shown by the higher percentages of T cells expressing IL-2, IFNγ or TNFα, particularly in the CD4^+^ T cell compartment (Fig. 4D). These data indicate that CBL0137 promotes the generation of tumor-specific T cell responses that can contribute to the observed therapeutic efficacy.

Local induction of ICD not only increases tumor cell immunogenicity, but also promotes the release of soluble mediators such as ATP, HMGB1 and other endogenous danger-associated molecular patterns (DAMPs) that trigger pro-inflammatory responses and promote recruitment and activation of immune cells [37]. To investigate the in vivo immunomodulatory effects of CBL0137 on the tumor microenvironment, we performed Opal multi-plex immunohistochemistry (IHC) and multi-parametric flow cytometry on tumors collected 10 days after the last CBL0137 treatment and characterized infiltration, activation and exhaustion profiles of T and NK cells in treated and untreated tumors [38]. Opal analysis revealed that CBL0137 reduced the relative number of CD8^+^ T cells and NK (NCR1^+^) cells per 1000 nuclei with no significant change in the proportion of total CD4^+^ T cells and Treg (CD4^+^ CD25^+^ FoxP3^+^) cells (Fig. S7A). In line with Opal staining results, flow cytometry profiling also showed a trend towards decreased percentage of T cells and NK cells in CBL0137-treated tumors compared to vehicle-treated tumors, although not statistically significant (Fig. S7B, S7C). In an independent experiment, CBL0137 induced a significant increase in the proportion of total intratumor myeloid cells mainly mediated by a significant increase in the proportion of Monocytic-MDSCs (CD11b^+^F4/80^-^Ly6G^-^Ly6C^high^) (Fig. S8A–D). A trend towards a higher proportion of PMN-MDSCs (CD11b^+^F4/80^-^Ly6G^+^Ly6C^low^) and a lower proportion of macrophages (CD11b^+^F4/80^high^) were also observed in tumors from CBL0137-treated mice. Of note, the drug was shown to reduce the level of IL-10 expression in PMN-MDSCs suggesting a less prominent immunosuppressive activity of these cells. Moreover, CBL0137-treated tumors showed a higher proportion of mature/activated and immunostimulatory PD-L1^+^ macrophages [39]. CBL0137-treated tumors also showed a significantly higher proportion of CD80^+^PD-L1^+^ macrophages characterized by reduced immunosuppressive function [40, 41]. Within the same experiment, we also observed that the proportion of intratumor B lymphocytes within CD45^+^ cells ( < 4%) was not altered by the drug. Moreover, the ratio between pre-B (CD19^low^CD45R^low^IGM^-^) immature (CD19^low^CD45Rl^ow^IGM^+^) or mature (CD19^high^CD45R^high^) B cells did not change in tumors from CBL0137-treated mice (Fig. S8E, F). These findings do not support a relevant role of B cells in mediating the anti-tumor activity of CBL0137.

### Activation and exhaustion profiling of T and NK cells

**Fig. 5:**
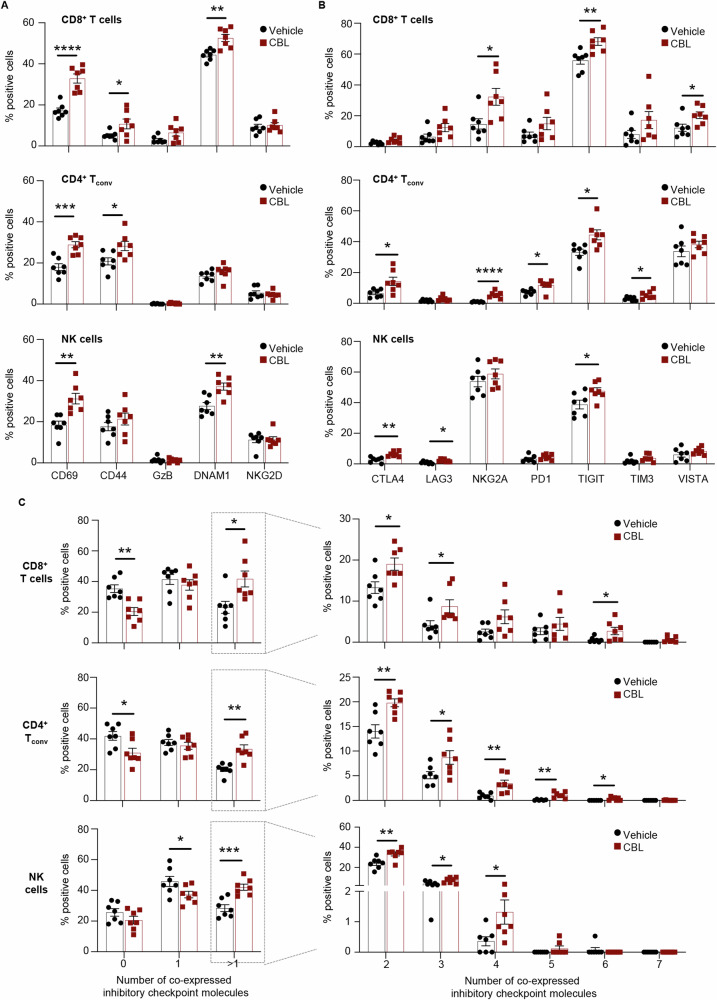
CBL0137 treatment induces activation and exhaustion of tumor infiltrating immune effector cells. Mice and tumors as described in Fig. 4A. Percentages of CD8^+^ T cells (top), CD4^+^ T_conv_ cells (middle) and NK cells (bottom) expressing the indicated activation markers (**A**) or inhibitory checkpoint molecules (**B**). Data are presented as mean ± SEM (*n* = 7 mice/group); *t* Test, *p < 0.05, **p < 0.01, ***p < 0.001****p < 0.0001. Outliers were excluded using Prism 9 and the recommended ROUT method with a False Discovery Rate (FDR) of Q = 0.5%. **C** Boolean gating analysis for the co-expression analysis of the 7 inhibitory checkpoint molecules shown in (B) in CD8^+^ T cells (top), CD4^+^ T_conv_ cells (middle) and NK cells (bottom). Left: percentages of cells co-expression 0, 1, or more then 1 ( > 1) inhibitory checkpoint molecule. Right: percentages of cells co-expressing any combination of 2, 3, 4, 5, 6 inhibitory checkpoint molecules, or all 7. Data are presented as mean ± SEM (*n* = 7 mice/group); *t* Test, *p < 0.05, **p < 0.01, ***p < 0.001. Outliers were excluded using Prism 9 and the recommended ROUT method with a False Discovery Rate (FDR) of Q = 0.5%.

Despite the apparent reduction in the relative number of effector cells, CBL0137 increased the proportions of cells expressing the early activation marker CD69 in all immune effector compartments e.g., CD3^+^CD8^+^ T cells, CD4^+^FOXP3^neg^ T_conv_ cells and CD3^neg^CD49b^+^ NK cells (Fig. 5A). CBL0137 also significantly increased the proportion of CD8^+^ T cells and CD4^+^ T_conv_ cells expressing the CD44 memory marker (Fig. 5A). Furthermore, a significant increase in CD8^+^ T cells and NK cells expressing the co-stimulatory and activation marker DNAM1 was also observed in treated tumors. Of note, the level of expression of DNAM1 was also significantly increased by CBL0137 in CD8^+^ T cells (Figs. S9A). All together, these data indicate activation of both adaptive and innate effector cells infiltrating CBL0137-treated tumors.

**Fig. 6:**
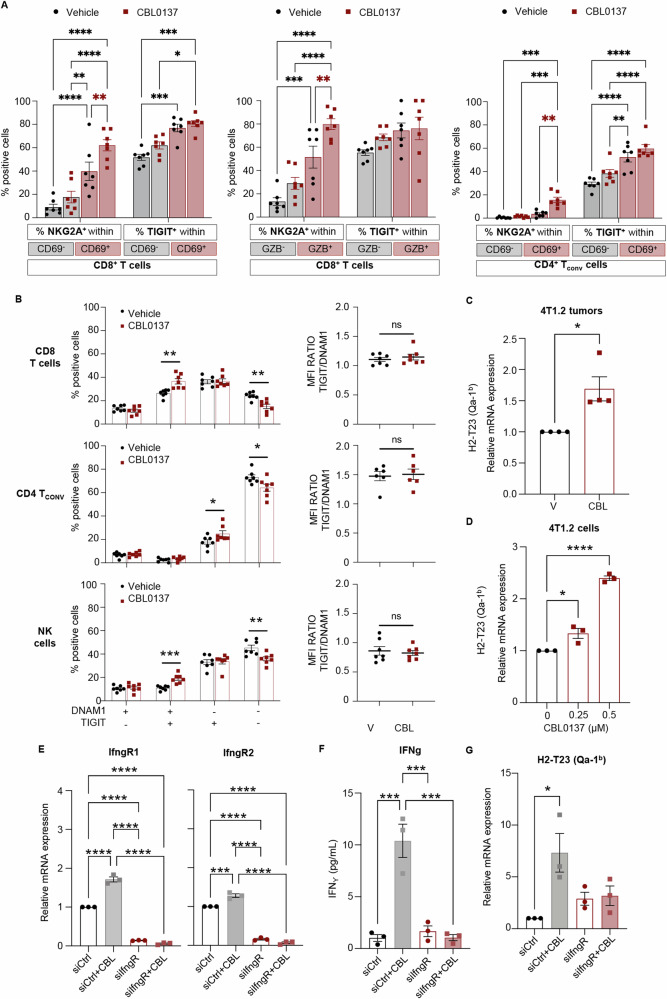
CBL0137 treatment induces the TIGIT/DNAM1 and NKG2A/Qa-1^b^ axes in 4T1.2 tumors. **A** Mice and tumors as described in Fig. 4A. Left: percentages of NKG2A or TIGIT positive cells within CD69 positive and negative CD8^+^ T cells in tumors from control (Vehicle) or treated mice (CBL0137). Center: percentages of NKG2A or TIGIT positive cells within GZB positive and negative CD8^+^ T cells in tumors from control (Vehicle) or treated mice (CBL0137). Right: percentages of NKG2A or TIGIT positive cells within CD69 positive and negative CD4^+^ T_conv_ cells in tumors from control (Vehicle) or treated mice (CBL0137). Data are presented as mean ± SEM (*n* = 7 mice/group); Two-way ANOVA with Tukey’s multiple comparisons test, *p < 0.05, **p < 0.01, ***p < 0.001****p < 0.0001. **B** Mice and tumors as described in Fig. 4A. Left: Percentages of DNAM1 and TIGIT double negative (DNAM1^-^TIGIT^-^), double positive (DNAM1^+^TIGIT^+^), and single positive (DNAM1^+^TIGIT^-^ or DNAM1^-^TIGIT^+^) cells in CD8^+^ T cells (top), CD4^+^ T_conv_ cells (middle) and NK cells (bottom). Right: Ratio between the expression levels (MFI) of TIGIT and DNAM1 on DNAM1^+^TIGIT^+^ double positive CD8^+^ T cells (top), CD4^+^ T_conv_ cells (middle) and NK cells (bottom). Data are presented as mean ± SEM (n = 7 mice/group); *t* Test. Outliers were excluded using Prism 9 and the recommended ROUT method with a False Discovery Rate (FDR) of Q = 0.5%. **C** Mice and tumors as in Fig. 2. Relative mRNA expression for the NKG2A ligand, Qa-1^b^, in CBL0137-treated and untreated (vehicle, V) 4T1.2 tumors. Data are presented as mean ± SEM (*n* = 4 mice/group); *t* Test, *p < 0.05. **D** Relative mRNA expression for the NKG2A ligand, Qa-1^b^, in 4T1.2 cells treated in vitro with CBL0137 (0-0.5 µM) for 48 h. Data are presented as mean ± SEM (*n* = 3); One-way ANOVA with Tukey’s multiple comparisons test, *p < 0.05, ****p < 0.0001. **E–G** 4T1.2 cells were transfected with either control siRNAs (siCtrl) or a mixture of IfngR1 and IfngR2-specific siRNAs (siIfngR) for 24 h, and then treated with 0.5 µM CBL0137 for 48 h or left untreated. **E** Relative mRNA levels for IfngR1, and IfngR2 analyzed by RT-qPCR. **F** IFNγ levels in the cell culture media analyzed by ELISA. **G** Relative mRNA levels for H2-T23 (Qa-1^b^) analyzed by RT-qPCR. Data are presented as mean ± SEM (*n* = 3); One-way ANOVA with Tukey’s multiple comparisons test, **p < 0.01, ***p < 0.001, ****p < 0.0001.

To investigate the extent of exhaustions of tumor infiltrating CD8^+^, CD4^+^ T_conv_ and NK cells, the expression of seven inhibitory immune checkpoints (CTLA4, LAG3, NKG2A, PD1, TIGIT, TIM3, and VISTA) was also analyzed within the same flow cytometry panel. We observed a consistent trend towards higher proportions of immune effector cells expressing inhibitory immune checkpoint molecules (ICMs) in CBL0137-treated tumors, with NKG2A and TIGIT being among the most frequently expressed in all effector compartments and upregulated by the treatment (Fig. 5B). Boolean gating was then used to evaluate the extent of co-expression of the seven ICMs in the three immune cell compartments. The progressive upregulation of these inhibitors determines the hierarchical loss of effector functions and acquisition of more dysfunctional/exhausted phenotypes. CBL0137 treatment resulted in a significant increase in the proportions of CD8^+^, CD4^+^ T_conv_ and NK cells co-expressing more than 1 ICM and even up to 6 ICMs (Fig. 5C). Boolean data were further interrogated to identify the most frequently represented ICM combinations in each immune cell compartment and those significantly increased by CBL0137 (Figs. S10A–S12A). As summarized in Figs. S10B–S12B, TIGIT and NKG2A were the ICMs most frequently represented in the CD8^+^ and NK cells cell subpopulations significantly expanded by CBL0137. In particular, all immune effector compartments infiltrating 4T1.2 tumors have high proportions of TIGIT^+^ cells, and consequently TIGIT is frequently found in combination with other ICMs (Figs. 5B, S10B–S12B). By contrast, despite the low proportions of NKG2A^+^ cells within CD8^+^ T cells, NKG2A was found in most subpopulations significantly expanded by CBL0137 (Fig. S10B–S12B). Moreover, the proportion of NKG2A^+^ cells was significantly higher in tumor infiltrating CD8^+^ T cells also expressing CD69^+^ or GZB^+^ as well as in CD69^+^ CD4^+^ T_conv_ cells (Figs. 6A, S13). Notably, enrichment in NKG2A^+^ cells was further enhanced by CBL0137 in these subpopulations (Fig. 6A; red asterisks). TIGIT^+^ cells were also enriched in activated cells, however to a lower extent which did not always reach statistical significance and was not further enhanced by CBL0137.

TIGIT is highly expressed on human and murine immune cells infiltrating several tumor types where it negatively regulates anti-tumor responses by binding CD155 or CD112, which are widely expressed on tumor cells. These same ligands are shared with DNAM1, which, on the contrary, promotes cytotoxicity and enhances anti-tumor responses. Therefore, when co-expressed on the same cell, the balance between these two receptors determines the shift between stimulatory and inhibitory responses. Figure 6B (left panel) shows the distribution of single and double positive cells for DNAM1 and TIGIT. In all three effector compartments (CD8^+^, CD4^+^ T_conv_ and NK cells), CBL0137 significantly reduced the proportion of DNAM1^neg^TIGIT^neg^ cells and significantly increases the proportion of DNAM1^+^TIGIT^+^ cells. On note, in all DNAM1^+^TIGIT^+^cells, no changes in the ratio between DNAM1 and TIGIT expression (MFI) was observed upon CBL0137 treatment (Fig. 6B; right panel). This data suggests that the resulting balance between activation and inhibition mediated by the TIGIT/DNAM1 axis might not be affected by CBL0137 treatment.

### CBL0137 upregulates Qa-1^b^ on tumor cells and synergizes with NKG2A blockade in inhibiting in vivo growth

NKG2A exerts its inhibitory effects by engaging with HLA-E (in humans) or Qa-1 (in mice) [42]. HLA-E protein levels are generally higher in tumor cells than in healthy tissues and available evidence indicates that IFNγ can upregulate HLA-E expression [43–45].

Given the robust interferon response induced by CBL0137 (Fig. 2), we examined if CBL0137 modulates Qa-1^b^ mRNA levels in 4T1.2 tumors in vivo. As shown in Fig. 6C, significantly higher Qa-1^b^ mRNA levels were observed in CBL0137-treated tumors compared to controls. These results were confirmed in vitro where treatment of 4T1.2 cells with CBL0137 also increased Qa-1^b^ mRNA levels in a dose-dependent manner (Fig. 6D). We next investigated whether upregulation of Qa-1^b^ was mediated by the increased levels of IFNγ induced by CBL0137 in these cells. 4T1.2 cells were transfected with either scrambled siRNAs (siCtrl) or a combination of siRNAs specific for IFNγ receptor 1 and 2 (siIfngR), and then treated with or without 0.5 µM CBL0137 for 24 h. Notably, CBL0137 treatment of siCtrl cells significantly upregulated (i) the mRNA levels of both IFN receptors (Fig. 6E) and (ii) the levels of secreted IFNγ (Fig. 6F). IFNγ receptor 1 and 2 knockdown, confirmed by RT-qPCR (Fig. 6E), abrogated the increase in IFNγ secretion induced by CBL0137 (Fig. 6F). This is consistent with the possible positive feedback loop sustaining the level of IFNγ production [43, 45]. Interestingly, while CBL0137 significantly increased Qa-1^b^ mRNA levels in siCtrl cells, it failed to increase Qa-1^b^ mRNA levels in IFNγ receptor knocked-down 4T1.2 cells (Fig. 6G). Taken together, our data indicate that CBL0137 increases Qa-1^b^ mRNA expression via IFNγ signaling.

**Fig. 7:**
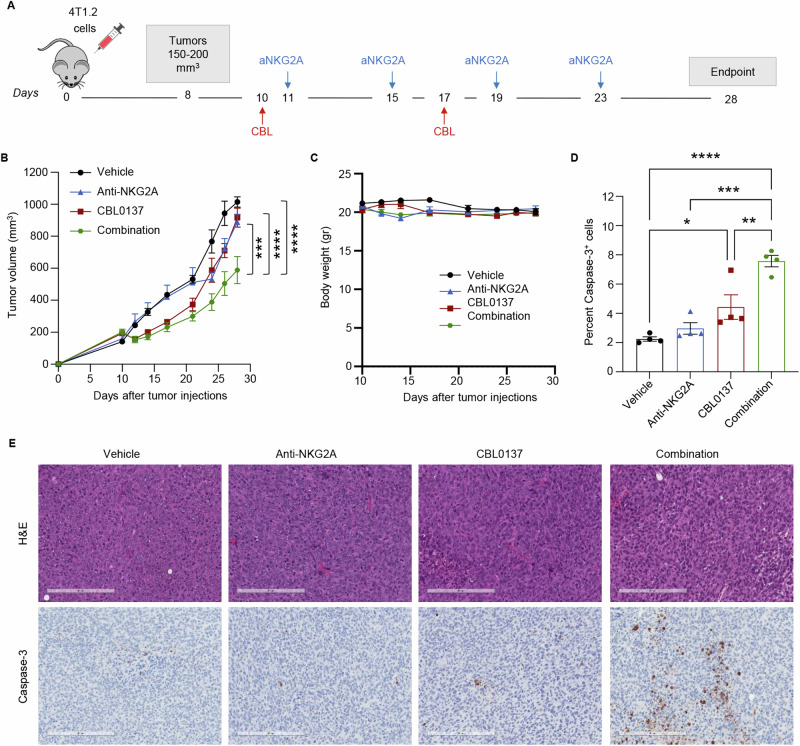
CBL0137 exerts a synergistic anti-cancer activity when combined with NKG2A blockade in the 4T1.2 model. **A** Balb/c mice were orthotopically injected with 4T1.2 cells and treated with either CBL0137 alone (60 mg/kg), or anti-NKG2A alone (200 µg), or their combination at the indicated days. Treatment commenced when tumors reached 150–200 mm^3^ in size to develop established tumors. Tumors were collected on day 28 for processing and IHC staining as described in methods. Tumor growth over time measured by caliper (**B**) and tumor volumes at day 28 (**C**) Data are presented as mean ± SEM (*n* = 6 mice/group); Two-way ANOVA with Sidak’s multiple comparisons test, ***p < 0.001, ****p < 0.0001. **D**, **E** Representative IHC images of H&E and ApopTag staining (Caspase-3) of primary 4T1.2 tumors treated with vehicle, CBL0137, anti-NKG2A blocking antibody, or the combination therapy for 2-weeks (**D**). Quantification of ApopTag staining in the primary 4T1.2 tumors. Percentage of Apoptotic cells is presented as mean ± SEM (*n* = 4 tumors/group) (**E**). One-way ANOVA with Sidak’s multiple comparisons test, *p < 0.05, **p < 0.01, ***p < 0.001, ****p < 0.0001.

Overall, these results suggest a potential role of NKG2A/Qa-1^b^ axis in hampering the activation of CD8^+^ and CD4^+^ T_conv_ cells. To exploit these results for therapeutic purposes, mice bearing 4T1.2 tumors were treated with CBL0137 in combination with anti-NKG2A antibody (Fig. 7A). As shown in Fig. 7B, C, the combination treatment significantly inhibited tumor growth when compared with untreated control mice or mice receiving single therapies. Notably, IHC analysis for cleaved Caspase-3 revealed that CBL0137 combined with anti-NKG2A significantly increased the percentage of apoptotic tumor cells compared to single agent treatments (Fig. 7D, E). These findings are consistent with an efficient cooperation between CBL0137 and NKG2A blockade in promoting the induction of therapeutically relevant anti-tumor immune responses in the 4T1.2 TNBC model.

## Discussion

Drugs able to directly inhibit tumor cell growth and indirectly reverse immune evasive mechanisms represent promising candidates for the development of novel combination therapies aimed at promoting efficient immunosurveillance and elimination of difficult to treat tumors, such as TNBCs. CBL0137, with its known ability to promote Type I interferon responses [20] and transcriptionally inhibit the oncogenic MYC pathway [22], may represent one of these candidate drugs.

Our data show that CBL0137 can significantly inhibit the growth of MYC-high TNBC cells by downregulating MYC transcription resulting in inhibition of its downstream target genes in an SSRP1 and p65 independent manner. This mechanism is different from that observed in other settings where CBL0137 exerted anticancer activity through SSRP1 and/or p65 [15, 17]. In immunocompromised mice, we confirmed a significant direct anti-proliferative activity of CBL0137 on MYC-high MDA-MB-231 TNBC tumors in vivo, where CBL0137 significantly reduced the growth of primary tumors.

Recent studies have shown that MYC can promote an immunosuppressive tumor microenvironment in multiple human cancers [24, 25, 46] and MYC overexpression correlated with low immune cell infiltration in TNBC patients [47]. Moreover, MYC activation was shown to repress the STING-IFN signaling in various tumor types, including TNBC, resulting in reduced immune cell infiltration and impaired innate anti-tumor immune responses (52–55). Consistent with these observations, our results indicate that CBL0137 significantly inhibited the growth of murine 4T1.2 tumors in immunocompetent Balb/c mice and to a much lower extent in immunocompromised RAG2 knockout Balb/c mice, further confirming the immunomodulatory role of the drug. Whole transcriptomic analysis on CBL0137-treated 4T1.2 tumors confirmed the ability of this drug to inhibit the expression of *MYC* and its downstream targets genes in vivo. Importantly, CBL0137 simultaneously upregulated the expression of genes belonging to the Type I IFN pathway and the levels of IFNγ in the serum of treated mice. These data are consistent with the known role played by *MYC* as a key negative regulator of the interferon pathway in cancer patients. MYC, together with its co-repressor MIZ1, was shown to suppress multiple interferon target genes by directly binding to their promoters in TNBCs [8].

Upregulation of immunomodulatory pathways as well as the requirement for an intact adaptive immune system to achieve optimal therapeutic efficacy, prompted us to investigate the possible enhanced immunogenicity in TNBC cells treated with CBL0137. Indeed, we demonstrate that CBL0137 induces apoptosis and ICD markers in vitro and that immunization with CBL0137-treated cells stimulates a partially protective immune response against the challenge with live 4T1.2 cells, supporting the increased immunogenicity of CBL0137-treated cells. The detection of increased proportions of tumor antigen-specific circulating T cells in 4T1.2-bearing mice treated with CBL0137 is consistent with the induction of ICD in vivo and with the ability of the drug to elicit therapeutically relevant anti-tumor immune responses. These results agree with the increased AH1 dextramer reactive CD8^+^ T cells observed in the CT26 model upon CBL0137 treatment [31]. In the same CT26 tumor model, one-week of CBL0137 treatment increased the number of infiltrating CD44^+^CD8^+^ effector T cells, [31]. Our high dimension flow cytometric profiling of CBL0137-treated 4T1.2 tumors extended these findings. In CBL0137-treated tumors, we observed an expansion of the myeloid compartment, mainly Monocytic-MDSCs, at the expense of T lymphocytes. However, the drug increased the proportion of intra-tumoral myeloid cells with reduced immunosuppressive activity (PMN-MDSCs with decreased expression of IL-10) or with more mature/activated phenotype and immunostimulatory function (PD-L1^+^ macrophages) [39]. Notably, CBL0137-treated mice also showed a significantly higher proportion of CD80^+^PD-L1^+^ macrophages. In these cells, PD-L1 can heterodimerize in *cis* with CD80 thereby preventing both PD-L1:PD1 and CD80:CTLA-4 interactions without interfering with the ability of CD80 to activate CD28 on T cells [40, 41]. Phenotypic profiling also showed that the percentages of CD69^+^, CD44^+^, and DNAM^+^ cells were significantly increased in all immune effector compartments analyzed (CD8^+^ T-cells, CD4^+^FoxP3^-^ T-cells, and NK cells), indicating that the drug enhances both adaptive and innate anti-tumor immune responses in the 4T1.2 TNBC model.

In response to prolonged activation and antigen exposure, immune cells can homeostatically upregulate the expression of inhibitory immune checkpoint molecules to dampen excessive inflammation. Tumors highjack these physiological mechanisms to evade immune surveillance. Supporting this notion, our immune profiling carried out on immune cells infiltrating CBL1037-treated tumors showed a significant upregulation of multiple inhibitory immune checkpoint receptors with significantly increased proportions of effector cells co-expressing multiple inhibitory checkpoint molecules, a known marker of functional exhaustion. Among those, TIGIT and NKG2A were the most represented in the exhausted effector cell populations expanded by CBL0137. DNAM1 and TIGIT share the same ligands [48], therefore an activating versus an inhibitory signaling outcome will depend on the DNAM1/TIGIT ratio which was, however, not altered by CBL0137. In contrast, CBL0137 significantly increased the proportions of NKG2A^+^ cells within all activated effector cell compartments, and, importantly, upregulated the expression of H2-T23 (Qa-1^b^), the ligand for mouse NKG2A, in 4T1.2 cells in vitro and in vivo. All together, these data suggest that the therapy-induced upregulation of NKG2A and Qa-1^b^ may prevent full exploitation of the immunomodulatory properties of CBL0137.

Activation of anti-tumor immune response by effector CD8^+^ T-cells and increased IFNγ levels have been shown to promote the expression of HLA-E, the human orthologue of mouse Qa-1^b^, in tumor cells [44, 45]. In line with these studies, our results indicate that suppression of IFNγ, via genetic depletion of the IFNγ receptors, abrogated CBL0137-induced Qa-1^b^ expression in 4T1.2 cells in vitro. The expression of HLA-E is increased in multiple human cancers including lung, pancreas, stomach, colon, head and neck, melanoma, and prostate cancers [49–52]. The HLA-E peptide complex is recognized by the CD94/NKG2A heterodimer receptor expressed on activated NK cells and on a subset of CD8^+^ T cells during viral infections and in tumors [49, 53–55]. In patients with invasive breast cancer, increased expression of the inhibitory receptor NKG2A was observed on NK cells and correlated with their decreased cytotoxic function [56]. In vivo NKG2A blockade using a monoclonal antibody was shown to enhance anti-tumor immunity by potentiating the cytotoxic effector functions of both NK and CD8^+^ T-cells in a syngeneic model of B-cell lymphoma [57]. Moreover, combined blockade of NKG2A and PD-L1 was recently shown to efficiently control the in vivo growth of MHC-I heterogeneous murine TNBC models [58]. Monalizumab, a humanized anti-NKG2A antibody, enhanced NK and CD8^+^ T-cells activity as well as the efficacy of Cetuximab in a phase II clinical trial with head and neck squamous cell carcinoma patients [57]. On these grounds and considering that our results clearly indicate that NKG2A is an attractive therapeutic target, we have investigated whether inhibition of NKG2A may enhance the therapeutic efficacy of CBL0137. Notably, the results presented herein demonstrate that the combination with NKG2A blockade enhanced the anti-cancer activity of CBL0137. These findings strengthen the relevance of a precise identification of the appropriate target checkpoint molecule(s) to rationally design effective combination therapies. It is worth considering that, in this study, NKG2A was chosen because it was specifically modulated by CBL0137. Further studies with human tumor samples are required to assess the reliability of the use of this criterion to select immune checkpoint inhibitors for combination immunotherapy. Notably, our findings also support the requirement of enhancing the generation of anti-tumor innate and adaptive immunity effectors using immunomodulatory drugs such as CBL0137 to increase the therapeutic efficacy of immune checkpoint-based immunotherapy, including anti-NKG2A antibodies.

CBL0137 has entered several clinical trials, including a completed phase Ib study of orally administered CBL0137 in patients with advanced solid tumors. Regression of up to 21% target lesions was documented in 4 patients, 2 of which were breast cancer patients [59]. Prospective clinical trials may elucidate whether NKG2A blockade can improve the therapeutic efficacy of CBL0137, and whether this novel combination therapy may serve as an effective treatment option for MYC-overexpressing TNBCs.

## Materials & methods

### Cell lines and reagents

All breast cancer cell lines were obtained from the American Type Culture Collection (ATCC). The 4T1.2 cell line was kindly provided by Dr Norman Pouliot, Olivia Newton-John Cancer Research Institute, Australia. All breast cancer cell lines were cultured in DMEM media supplemented with 10% fetal bovine serum (FBS). All cell lines were tested for Mycoplasma infection and authenticated using short tandem repeat (STR) profiling by scientific services at QIMR Berghofer Medical Research Institute. CBL0137 was purchased from Selleck Chemicals (Cat #: S8483). Anti-NKG2A blocking antibody was purchased from BioLegend (Cat #: BE0321). The list of antibodies and RT-qPCR primers used in this study is provided in Table S1, S2, respectively.

### Immunogenic cell death assays

#### In vitro

Breast cancer cells were seeded in T25 flasks until ~70% of confluence. The cell culture media was then replaced with 5 mL of complete media containing the indicated dose of CBL0137 (0–2.5 µM) and incubated at 37 °C, 5% CO_2_ for 24 h. At the end of incubation, cells were harvested and stained with Pacific Blue-Annexin V (Biolegend, Cat # 640918), 7-AAD (Biolegend, Cat# 420404) and anti-Calreticulin-AF647 (Abcam, Cat # ab196159). Samples were acquired using an LSR-FortessaX20 Flow Cytometer and analyzed using FlowJo software.

#### In vivo

4T1.2 cells were seeded in T25 flasks until ~70% confluence. The cell culture media was then replaced with 5 mL of complete media containing 5 µM CBL0137 for 24 h. 100,000 CBL0137-treated cells (80–90% apoptotic cells) or killed with 3 cycles of freezing and thawing, were injected into the 4th mammary fat-pad of female Balb/c mice. Four days later, mice were re-challenged with untreated live 4T1.2 cells by injection into the 9th mammary fat-pad and mice were monitored for tumor growth.

### In vivo animal models

#### Human MDA-MB-231 orthotopic model

In total, 3 × 10^6^ MDA-MB-231 cells were prepared in 50% growth factor-reduced Matrigel (BD, Biosciences, Bedford, USA)/PBS and injected into the right 4th inguinal mammary fat pad of 6-weeks old female immunocompromised NOD/SCID mice.

#### Human MDA-MB-231 experimental brain metastasis

Brain-seeking MDA-MB-231-Br cells (25,000 cells) were prepared in 1X PBS and injected intracranially into the lateral portion of the striatum or cerebellum of 6-weeks old female Balb/c Nude mice. Stereotactic coordinates for striatum injections were 1.6 mm posterior to the bregma, 0.8 mm lateral (right) of the midline, and 3 mm deep from the dura.

#### Murine 4T1.2 orthotopic model

In total, 1 × 10^5^ 4T1.2 cells resuspended in 1X PBS were injected into the right 4th inguinal mammary fat pad of 6-weeks old female immunocompetent Balb/c mice and 6-weeks old female RAG2 knockout Balb/c mice.

Once tumor size reached ~30–50 mm^3^, mice were randomized blindly into different treatment groups and were then i.v. injected with the vehicle or CBL0137 (60 mg/kg) once per week for 2–3 weeks. Tumor growth was measured thrice weekly using a digital caliper. For CBL0137 and anti-NKG2A antibody combination therapy, 4T1.2 tumor-bearing Balb/c mice were randomized into four treatment groups when tumor size reached ~150–200 mm^3^ (established tumors): (i) vehicle + Control IgGs (200 µg twice per week, i.p.), (ii) CBL0137 (60 mg/kg, once per week, i.v.), (iii) anti-NKG2A antibody (200 µg, twice per week, i.p.), and (iv) combination treatment. Mice were treated for two weeks. For all orthotopic models, the tumor volume was calculated using the following formula: tumor volume = [LxW^2^]/2, where W = width of the tumor and L = length of the tumor. For the intracranial brain metastases model, tumor growth was monitored once per week by bioluminescence imaging (IVIS Xenogen) where nice were i.p. injected with 150 mg/kg of VivoGlo^TM^ Luciferin (Promega) 10 min before imaging.

### RNA Seq

Murine 4T1.2 cells were injected into 6-weeks old female Balb/c mice as describe above. Once tumor size reached ~30–50 mm^3^, mice were randomized blindly into different treatment groups and were then treated with the vehicle or CBL0137 (60 mg/kg, once per week, i.v.) for one week (*n* = 4). Tumors were harvested after one week of drug treatment. Total RNA was extracted using the RNeasy Plus Mini Kit (Qiagen) per manufacturer’s instruction. Strand-specific RNA-seq was performed by BGI Genomics using the polyA-selection method. Libraries were constructed and sequenced on the G-400 platform (BGI Group) with 2 × 100 bp reads sequenced to a minimum of 20 million read pairs per sample. Sequence reads were trimmed for adapter sequences using Cutadapt version 1.9 [60] and aligned using STAR version 2.5.2a [61] to the mouse genome version mm10. Quality control metrics were computed using RNA-SeQC version 1.1.8 [62] and transcripts were quantified using RSEM version 1.2.30 [63]. All downstream RNA-seq analysis was performed using R version 3.6.2 [64]. Differential expression analysis was conducted using the R package edgeR and genes were filtered out prior to differential gene expression analysis if the counts per million was less than 1 in more than half of the samples. Genes were considered statistically differentially expressed if genes had a false discovery rate < 0.05 and a fold-change ≥|2 | . Mouse genes were converted to human gene homologs using the R package biomaRt. Significant differentially expressed genes were analyzed for gene ontology enrichment analysis using DAVID. Pre-ranked gene set enrichment analysis was also performed using MSigDB v7.1.

### Polyfunctional T cell assay

Blood was collected from mice into EDTA treated tubes at indicated the time point and lysed by ACK (150 mM NH_4_Cl, 10 mM KHCO_3_, 0.1 nM Na_2_EDTA) buffer to exclude erythrocytes. After washing with complete RPMI medium (Gibco), white blood cells were plated in round bottom 96-well plates at a density of 1 × 10^6^ cells/well and ex vivo stimulated with a pool of epitopes (mSurvivin53-67, mSurvivin66-74 and mTERT167-175, 10 µg/ml/epitope) for 6 h. Brefeldin A was added after 30 min to block cytokine secretion. At the end of incubation, cells were surface stained with anti-CD3e, anti-CD4, anti-CD8, then intracellularly stained with anti-TNFα, anti-IFNγ and anti-IL2 following fixation and permeabilizing with the eBioscience^TM^ FOxp3/Transcription factor staining buffer according to the Manufacturer instructions. Samples were acquired using an LSR-FortessaX20 Flow Cytometer. Cytokine co-expression profiles were quantified using the Boolean gating function of FlowJo software. The reagents used for the polyfunctional T cell assay are listed in Table S3.

### Flow cytometry on tumors

Tumors (~1 g) were mechanically minced, resuspended in digestion buffer (RPMI with 10% FBS, 0.1 mg/mL collagenase D and 0.1 μg/mL DNase) and incubated for 30 min at 37°C in agitation. Samples were then dissociated by pipetting up and down and filtered through a 70 μm cell strainer into a 50 mL conical tube. Cells were then pelleted and resuspended in ACK buffer to remove erythrocytes. Cells were then washed, resuspended in PBS and stained for surface and intracellular markers as previously described [38]. The list of the antibodies and reagents used for FACS profiling are indicated in Table S4.

### Interferon-gamma ELISA

4T1.2 tumor-bearing mice were treated either with vehicle or CBL0137 (60 mg/kg, once per week, i.v.) for one week. After one week of treatment, mice were euthanized, blood was collected, and serum was harvested from 4 mice per treatment group. IFNγ levels in the serum of vehicle-treated and CBL0137-treated mice were analyzed using ELISA MAX™ Deluxe Set Mouse IFNγ kit (BioLegend) as per manufacturer’s guidelines. For the quantification of IFNγ in the cell culture media, 4T1.2 cells were transfected with scramble, IfngR1, or IfngR2-specific siRNAs for 24 h, subsequently treated with CBL0137 (0.5 µM) for 24 h, and media was collected and diluted. IFNγ levels in the cell culture media were analyzed using ELISA MAX™ Deluxe Set Mouse IFNγ kit (BioLegend) as per manufacturer’s guidelines.

### Opal monoplex and multiplex immunofluorescence

#### Tissue preparation for staining

Slides were baked at 58 °C for 60 min before being de-paraffinized and rehydrated in a Gemini AS Automated Slide Stainer. Briefly, slides were placed in xylene for 5 min x2, 100% ethanol 1 min x3, 70% ethanol 1 min and distilled water for 1 min, followed by a 10 min wash in distilled water. TMA slides were prepared and stained using previously published methods with minor modifications [65].

#### Chromogenic monoplex IHC

Murine TNBC tumor samples were used for antibody and multiplex optimization. Antibodies used in this study are summarised in Table S6. Chromogenic immunohistochemistry using 3,3′‐diaminobenzidine (DAB) detection (BOND Polymer Refine Detection) was carried out to determine the primary antibody staining conditions and the order of primary antibody staining in the multiplex protocol. Initial antigen retrieval was performed in a Decloaking Chamber^TM^ NxGen at 110 °C for 5 min in citrate pH 6 or EDTA pH 9 antigen retrieval buffers. Staining was performed on a BOND RX automated immunostainer using BOND Epitope Retrieval (ER) Solutions: citrate‐based pH 6.0 ER1 or EDTA‐based pH 9.0 ER2.

#### Monoplex and multiplex immunofluorescence

Opal 6-plex detection kits (Akoya Biosciences, USA) were used for immunofluorescence staining, a tyramide signal amplification-based OPAL multiplexing technology. Primary antibody conditions and the order of antibody staining determined in the initial DAB optimization step were applied to both OPAL monoplex and multiplex optimisation. Each antibody was paired with an individual Opal fluorophore, with pairing based on the estimated abundance of the biomarker and the expected levels of biomarker co-expression within sample tissue. See Table S6 for further information on antibody conditions used in immunofluorescence staining. For each antibody, slides were blocked with an antibody diluent, followed by incubation of the primary antibody. Slides were then washed in wash buffer before being incubated with an Opal polymer horseradish peroxidase. Signal amplification was then performed by adding the Opal fluorophore, followed by stripping of the antibody. Staining quality and intensity of each biomarker was assessed against the Chromogenic IHC during optimisation of OPAL monoplex. Captured monoplex images were unmixed and analyzed with INFORM software version 2.5.1.

#### Image acquisition

DAB chromogenic stained slides were imaged on an AperioXT microscope using ×40 magnification. The quality of antibody labeling was reviewed before proceeding with OPAL multiplexing. Fluorescently labeled slides were imaged on a Vectra Polaris using ×20 magnification and auto-estimated exposure times. Regions were then selected for multispectral field scans and imaged using Phenochart software version 1.1.0. Images were unmixed using INFORM tissue analysis software version 2.5.1. Images were then stitched together in QuPath v0.2.3 using a script to produce a whole-slide multichannel, pyramidal OME-TIFF image.

#### Digital image analysis

Image analysis was performed using QuPath v0.2.3. Cell segmentation was created using the StarDist nucleus detection script using previously published methods [66]. Phenotyping of all biomarkers was created by generating object classifiers. Object classification was achieved via machine learning such as a random forest algorithm. Each classifier was thoroughly trained on multiple cell measurements and then merged into a composite classifier which was applied to all slides. Results are presented as relative number of positive cells per 1000 nuclei = [(number of immune cells/total number of nuclei detected in tumor)*1000].

### Immunohistochemistry

Immunohistochemistry analysis on primary 4T1.2 tumors treated with CBL0137 alone, anti-NKG2A alone, or the combination therapy was performed as described previously [67]. ApopTag staining was performed using the ApopTag peroxidase in situ apoptosis detection kit (S7100; Millipore-Sigma, Billerica, MA, USA).

### Statistical analysis

A pragmatic sample size of 5 or 6 mice per treatment group was chosen to allow adequate calculation of parameter estimates whilst being feasible [68]. No formal statistical power calculations were performed but our sample sizes are similar to those reported in previous publications [29, 69]. No exclusion has been made in this study. In vitro experiments were performed with at least three biological replicates unless otherwise described in figure legends and repeated in independent experiments. Statistics were performed on values from independent experiments. All values are presented as mean ± SEM. Data were analyzed using GraphPad Prism 9 (GraphPad Software, CA, USA). Statistical significance was determined by *t* Test when comparing two groups only or by ANOVA for multiple comparisons. Specific post-tests were applied with ANOVA as indicated in each figure legend. Data distribution was assumed to be normal, but this was not fully tested. Error bars represent the SEM and are described in the figure legends.

## Supplementary information

Supplementary materials and methods

Supplementary data file - RNA seq data file

## Data Availability

The datasets used and/or analyzed during the current study are available from the corresponding author on reasonable request. The materials used in this study are available from the corresponding author upon request. The RNA sequencing dataset supporting the conclusions of this article is available in the [The European Nucleotide Archive (ENA)] repository, [PRJEB63628]. The RNA sequencing analyzed data supporting the conclusions of this article are included within the article and its additional files.

## References

[1] Watase C, Shiino S, Shimoi T, Noguchi E, Kaneda T, Yamamoto Y, et al. Breast cancer brain metastasis-overview of disease state, treatment options and future perspectives. Cancers (Basel) 2021;13:1078. 10.3390/cancers13051078 PMC7959316 33802424

[2] Dong Y, Tu R, Liu H, Qing G. Regulation of cancer cell metabolism: oncogenic MYC in the driver’s seat. Signal Transduct Target Ther. 2020;5:124. 32651356 10.1038/s41392-020-00235-2 PMC7351732

[3] Felsher DW, Bishop JM. Reversible tumorigenesis by MYC in hematopoietic lineages. Mol Cell. 1999;4:199–207. 10488335 10.1016/s1097-2765(00)80367-6

[4] Jain M, Arvanitis C, Chu K, Dewey W, Leonhardt E, Trinh M, et al. Sustained loss of a neoplastic phenotype by brief inactivation of MYC. Science. 2002;297:102–4. 12098700 10.1126/science.1071489

[5] Shachaf CM, Kopelman AM, Arvanitis C, Karlsson A, Beer S, Mandl S, et al. MYC inactivation uncovers pluripotent differentiation and tumour dormancy in hepatocellular cancer. Nature. 2004;431:1112–7. 15475948 10.1038/nature03043

[6] Dang CV. MYC on the path to cancer. Cell. 2012;149:22–35. 22464321 10.1016/j.cell.2012.03.003 PMC3345192

[7] Dhanasekaran R, Baylot V, Kim M, Kuruvilla S, Bellovin DI, Adeniji N, et al. MYC and Twist1 cooperate to drive metastasis by eliciting crosstalk between cancer and innate immunity. eLife. 2020;9:e50731. 10.7554/eLife.50731 PMC6959993 31933479

[8] Zimmerli D, Brambillasca CS, Talens F, Bhin J, Linstra R, Romanens L, et al. MYC promotes immune-suppression in triple-negative breast cancer via inhibition of interferon signaling. Nat Commun. 2022;13:6579. 36323660 10.1038/s41467-022-34000-6 PMC9630413

[9] Choi SK, Hong SH, Kim HS, Shin CY, Nam SW, Choi WS, et al. JQ1, an inhibitor of the epigenetic reader BRD4, suppresses the bidirectional MYC-AP4 axis via multiple mechanisms. Oncol Rep. 2016;35:1186–94. 26573731 10.3892/or.2015.4410

[10] Delmore JE, Issa GC, Lemieux ME, Rahl PB, Shi J, Jacobs HM, et al. BET bromodomain inhibition as a therapeutic strategy to target c-Myc. Cell. 2011;146:904–17. 21889194 10.1016/j.cell.2011.08.017 PMC3187920

[11] Han H, Jain AD, Truica MI, Izquierdo-Ferrer J, Anker JF, Lysy B, et al. Small-molecule MYC inhibitors suppress tumor growth and enhance immunotherapy. Cancer Cell. 2019;36:483–497.e415. 31679823 10.1016/j.ccell.2019.10.001 PMC6939458

[12] Braso-Maristany F, Filosto S, Catchpole S, Marlow R, Quist J, Francesch-Domenech E, et al. PIM1 kinase regulates cell death, tumor growth and chemotherapy response in triple-negative breast cancer. Nat Med. 2016;22:1303–13. 27775704 10.1038/nm.4198 PMC5552044

[13] Wyce A, Ganji G, Smitheman KN, Chung CW, Korenchuk S, Bai Y, et al. BET inhibition silences expression of MYCN and BCL2 and induces cytotoxicity in neuroblastoma tumor models. PLoS One. 2013;8:e72967. 24009722 10.1371/journal.pone.0072967 PMC3751846

[14] Burkhart C, Fleyshman D, Kohrn R, Commane M, Garrigan J, Kurbatov V, et al. Curaxin CBL0137 eradicates drug resistant cancer stem cells and potentiates efficacy of gemcitabine in preclinical models of pancreatic cancer. Oncotarget. 2014;5:11038–53. 25402820 10.18632/oncotarget.2701 PMC4294371

[15] Gasparian AV, Burkhart CA, Purmal AA, Brodsky L, Pal M, Saranadasa M, et al. Curaxins: anticancer compounds that simultaneously suppress NF-kappaB and activate p53 by targeting FACT. Sci Transl Med. 2011;3:95ra74. 21832239 10.1126/scitranslmed.3002530 PMC6281439

[16] Barone TA, Burkhart CA, Safina A, Haderski G, Gurova KV, Purmal AA, et al. Anticancer drug candidate CBL0137, which inhibits histone chaperone FACT, is efficacious in preclinical orthotopic models of temozolomide-responsive and -resistant glioblastoma. Neuro-Oncol. 2017;19:186–96. 27370399 10.1093/neuonc/now141 PMC5604960

[17] Carter DR, Murray J, Cheung BB, Gamble L, Koach J, Tsang J, et al. Therapeutic targeting of the MYC signal by inhibition of histone chaperone FACT in neuroblastoma. Sci Transl Med. 2015;7:312ra176. 26537256 10.1126/scitranslmed.aab1803 PMC6207083

[18] Nesher E, Safina A, Aljahdali I, Portwood S, Wang ES, Koman I, et al. Role of chromatin damage and chromatin trapping of FACT in mediating the anticancer cytotoxicity of DNA-binding small-molecule drugs. Cancer Res. 2018;78:1431–43. 29339544 10.1158/0008-5472.CAN-17-2690 PMC5856628

[19] Wang J, Sui Y, Li Q, Zhao Y, Dong X, Yang J, et al. Effective inhibition of MYC-amplified group 3 medulloblastoma by FACT-targeted curaxin drug CBL0137. Cell Death Dis. 2020;11:1029. 33268769 10.1038/s41419-020-03201-6 PMC7710710

[20] Leonova K, Safina A, Nesher E, Sandlesh P, Pratt R, Burkhart C, et al. TRAIN (Transcription of Repeats Activates INterferon) in response to chromatin destabilization induced by small molecules in mammalian cells. eLife 2018;7:e30842. 10.7554/eLife.30842 PMC5815852 29400649

[21] Chang HW, Nizovtseva EV, Razin SV, Formosa T, Gurova KV, Studitsky VM. Histone chaperone FACT and curaxins: effects on genome structure and function. J Cancer Metastasis Treat 2019;5:78. 10.20517/2394-4722.2019.31 PMC6919649 31853507

[22] Kantidze OL, Luzhin AV, Nizovtseva EV, Safina A, Valieva ME, Golov AK, et al. The anti-cancer drugs curaxins target spatial genome organization. Nat Commun. 2019;10:1441. 30926878 10.1038/s41467-019-09500-7 PMC6441033

[23] Casey SC, Baylot V, Felsher DW. The MYC oncogene is a global regulator of the immune response. Blood. 2018;131:2007–15. 29514782 10.1182/blood-2017-11-742577 PMC5934797

[24] Casey SC, Tong L, Li Y, Do R, Walz S, Fitzgerald KN, et al. MYC regulates the antitumor immune response through CD47 and PD-L1. Science. 2016;352:227–31. 26966191 10.1126/science.aac9935 PMC4940030

[25] Kortlever RM, Sodir NM, Wilson CH, Burkhart DL, Pellegrinet L, Brown Swigart L, et al. Myc cooperates with Ras by programming inflammation and immune suppression. Cell. 2017;171:1301–1315.e1314. 29195074 10.1016/j.cell.2017.11.013 PMC5720393

[26] Bernards R, Dessain SK, Weinberg RA. N-myc amplification causes down-modulation of MHC class I antigen expression in neuroblastoma. Cell. 1986;47:667–74. 3096575 10.1016/0092-8674(86)90509-x

[27] Versteeg R, Noordermeer IA, Kruse-Wolters M, Ruiter DJ, Schrier PI. c-myc down-regulates class I HLA expression in human melanomas. EMBO J. 1988;7:1023–9. 3402430 10.1002/j.1460-2075.1988.tb02909.x PMC454430

[28] Escobar G, Moi D, Ranghetti A, Ozkal-Baydin P, Squadrito ML, Kajaste-Rudnitski A, et al. Genetic engineering of hematopoiesis for targeted IFN-alpha delivery inhibits breast cancer progression. Sci Transl Med. 2014;6:217ra213. 10.1126/scitranslmed.3006353 24382895

[29] Raninga PV, Lee A, Sinha D, Dong LF, Datta KK, Lu X, et al. Marizomib suppresses triple-negative breast cancer via proteasome and oxidative phosphorylation inhibition. Theranostics. 2020;10:5259–75. 32373211 10.7150/thno.42705 PMC7196287

[30] Eckhardt BL, Parker BS, van Laar RK, Restall CM, Natoli AL, Tavaria MD, et al. Genomic analysis of a spontaneous model of breast cancer metastasis to bone reveals a role for the extracellular matrix. Mol Cancer Res. 2005;3:1–13. 15671244

[31] Chen M, Brackett CM, Burdelya LG, Punnanitinont A, Patnaik SK, Matsuzaki J, et al. Stimulation of an anti-tumor immune response with “chromatin-damaging” therapy. Cancer Immunol, Immunother : CII. 2021;70:2073–86. 33439292 10.1007/s00262-020-02846-8 PMC8726059

[32] Arvindam US, van Hauten PMM, Schirm D, Schaap N, Hobo W, Blazar BR, et al. A trispecific killer engager molecule against CLEC12A effectively induces NK-cell mediated killing of AML cells. Leukemia. 2021;35:1586–96. 33097838 10.1038/s41375-020-01065-5 PMC8189652

[33] Fein MR, He XY, Almeida AS, Bruzas E, Pommier A, Yan R, et al. Cancer cell CCR2 orchestrates suppression of the adaptive immune response. J Exp Med. 2020;217:e20181551. 10.1084/jem.20181551 PMC7537399 32667673

[34] Kiss M, Vande Walle L, Saavedra PHV, Lebegge E, Van Damme H, Murgaski A, et al. IL1beta promotes immune suppression in the tumor microenvironment independent of the inflammasome and gasdermin D. Cancer Immunol Res. 2021;9:309–23. 33361087 10.1158/2326-6066.CIR-20-0431

[35] Kim DY, Pyo A, Yun M, Thangam R, You SH, Zhang Y, et al. Imaging calreticulin for early detection of immunogenic cell death during anticancer treatment. J Nucl Med. 2021;62:956–60. 33509975 10.2967/jnumed.120.245290 PMC8882882

[36] Chen R, Zou J, Zhong X, Li J, Kang R, Tang D. HMGB1 in the interplay between autophagy and apoptosis in cancer. Cancer Lett. 2024;581:216494. 38007142 10.1016/j.canlet.2023.216494

[37] Kroemer G, Galluzzi L, Kepp O, Zitvogel L. Immunogenic cell death in cancer therapy. Annu Rev Immunol. 2013;31:51–72. 23157435 10.1146/annurev-immunol-032712-100008

[38] Moi D, Zeng B, Minnie SA, Bhatt R, Wood J, Sester DP, et al. Multiparametric flow cytometry to characterize vaccine-induced polyfunctional T cell responses and T cell/NK cell exhaustion and memory phenotypes in mouse immuno-oncology models. Front Immunol. 2023;14:1127896. 37090730 10.3389/fimmu.2023.1127896 PMC10115975

[39] Wang L, Guo W, Guo Z, Yu J, Tan J, Simons DL, et al. PD-L1-expressing tumor-associated macrophages are immunostimulatory and associate with good clinical outcome in human breast cancer. Cell Rep. Med. 2024;5:101420. 38382468 10.1016/j.xcrm.2024.101420 PMC10897617

[40] Chaudhri A, Xiao Y, Klee AN, Wang X, Zhu B, Freeman GJ. PD-L1 binds to B7-1 only In Cis on the same cell surface. Cancer Immunol Res. 2018;6:921–9. 29871885 10.1158/2326-6066.CIR-17-0316 PMC7394266

[41] Zhao Y, Lee CK, Lin CH, Gassen RB, Xu X, Huang Z, et al. PD-L1:CD80 cis-heterodimer triggers the co-stimulatory receptor CD28 while repressing the inhibitory PD-1 and CTLA-4 pathways. Immunity. 2019;51:1059–1073.e1059. 31757674 10.1016/j.immuni.2019.11.003 PMC6935268

[42] van Hall T, Andre P, Horowitz A, Ruan DF, Borst L, Zerbib R, et al. Monalizumab: inhibiting the novel immune checkpoint NKG2A. J Immunother Cancer. 2019;7:263. 31623687 10.1186/s40425-019-0761-3 PMC6798508

[43] Dubrot J, Du PP, Lane-Reticker SK, Kessler EA, Muscato AJ, Mehta A, et al. In vivo CRISPR screens reveal the landscape of immune evasion pathways across cancer. Nat Immunol. 2022;23:1495–506. 36151395 10.1038/s41590-022-01315-x

[44] Gustafson KS, Ginder GD. Interferon-gamma induction of the human leukocyte antigen-E gene is mediated through binding of a complex containing STAT1alpha to a distinct interferon-gamma-responsive element. J Biol Chem. 1996;271:20035–46. 8702722 10.1074/jbc.271.33.20035

[45] Malmberg KJ, Levitsky V, Norell H, de Matos CT, Carlsten M, Schedvins K, et al. IFN-gamma protects short-term ovarian carcinoma cell lines from CTL lysis via a CD94/NKG2A-dependent mechanism. J Clin Investig. 2002;110:1515–23. 12438449 10.1172/JCI15564 PMC151808

[46] Muthalagu N, Monteverde T, Raffo-Iraolagoitia X, Wiesheu R, Whyte D, Hedley A, et al. Repression of the type I interferon pathway underlies MYC- and KRAS-dependent evasion of NK and B cells in pancreatic ductal adenocarcinoma. Cancer Discov. 2020;10:872–87. 32200350 10.1158/2159-8290.CD-19-0620 PMC7611248

[47] Xiao Y, Ma D, Zhao S, Suo C, Shi J, Xue MZ, et al. Multi-omics profiling reveals distinct microenvironment characterization and suggests immune escape mechanisms of triple-negative breast cancer. Clin Cancer Res. 2019;25:5002–14. 30837276 10.1158/1078-0432.CCR-18-3524

[48] Shibuya A, Shibuya K. DNAM-1 versus TIGIT: competitive roles in tumor immunity and inflammatory responses. Int Immunol. 2021;33:687–92. 34694361 10.1093/intimm/dxab085

[49] Gooden M, Lampen M, Jordanova ES, Leffers N, Trimbos JB, van der Burg SH, et al. HLA-E expression by gynecological cancers restrains tumor-infiltrating CD8(+) T lymphocytes. Proc Natl Acad Sci USA. 2011;108:10656–61. 21670276 10.1073/pnas.1100354108 PMC3127933

[50] Talebian Yazdi M, van Riet S, van Schadewijk A, Fiocco M, van Hall T, Taube C, et al. The positive prognostic effect of stromal CD8+ tumor-infiltrating T cells is restrained by the expression of HLA-E in non-small cell lung carcinoma. Oncotarget. 2016;7:3477–88. 26658106 10.18632/oncotarget.6506 PMC4823121

[51] van Esch EM, Tummers B, Baartmans V, Osse EM, Ter Haar N, Trietsch MD, et al. Alterations in classical and nonclassical HLA expression in recurrent and progressive HPV-induced usual vulvar intraepithelial neoplasia and implications for immunotherapy. Int J Cancer. 2014;135:830–42. 24415578 10.1002/ijc.28713

[52] van Montfoort N, Borst L, Korrer MJ, Sluijter M, Marijt KA, Santegoets SJ, et al. NKG2A blockade potentiates CD8 T cell immunity induced by cancer vaccines. Cell. 2018;175:1744–1755.e1715. 30503208 10.1016/j.cell.2018.10.028 PMC6354585

[53] Moser JM, Gibbs J, Jensen PE, Lukacher AE. CD94-NKG2A receptors regulate antiviral CD8(+) T cell responses. Nat Immunol. 2002;3:189–95. 11812997 10.1038/ni757

[54] Rapaport AS, Schriewer J, Gilfillan S, Hembrador E, Crump R, Plougastel BF, et al. The inhibitory receptor NKG2A sustains virus-specific CD8(+) T cells in response to a lethal poxvirus infection. Immunity. 2015;43:1112–24. 26680205 10.1016/j.immuni.2015.11.005 PMC4745883

[55] Sheu BC, Chiou SH, Lin HH, Chow SN, Huang SC, Ho HN, et al. Up-regulation of inhibitory natural killer receptors CD94/NKG2A with suppressed intracellular perforin expression of tumor-infiltrating CD8+ T lymphocytes in human cervical carcinoma. Cancer Res. 2005;65:2921–9. 15805295 10.1158/0008-5472.CAN-04-2108

[56] Mamessier E, Sylvain A, Thibult ML, Houvenaeghel G, Jacquemier J, Castellano R, et al. Human breast cancer cells enhance self tolerance by promoting evasion from NK cell antitumor immunity. J Clin Investig. 2011;121:3609–22. 21841316 10.1172/JCI45816 PMC3171102

[57] Andre P, Denis C, Soulas C, Bourbon-Caillet C, Lopez J, Arnoux T, et al. Anti-NKG2A mAb is a checkpoint inhibitor that promotes anti-tumor immunity by unleashing both T and NK cells. Cell. 2018;175:1731–1743.e1713. 30503213 10.1016/j.cell.2018.10.014 PMC6292840

[58] Taylor BC, Sun X, Gonzalez-Ericsson PI, Sanchez V, Sanders ME, Wescott EC, et al. NKG2A is a therapeutic vulnerability in immunotherapy resistant MHC-I heterogeneous triple negative breast cancer. Cancer Discov 2024;14:290–307. 10.1158/2159-8290.CD-23-0519 PMC10850946 37791898

[59] Fedyanin M, Tryakin A, Lisyanskaya AS, Solovyeva E, Fadeeva N, Gladkov O, et al. Results of a completed first-in-human phase Ib dose-escalation study of oral CBL0137 in patients with advanced solid tumors. J Clin Oncol. 2020;38:3607–3607.

[60] Martin M. Cutadapt removes adapter sequences from high-throughput sequencing reads. EMBnetjournal. 2011;17:10–12.

[61] Dobin A, Davis CA, Schlesinger F, Drenkow J, Zaleski C, Jha S, et al. STAR: ultrafast universal RNA-seq aligner. Bioinformatics. 2013;29:15–21. 23104886 10.1093/bioinformatics/bts635 PMC3530905

[62] DeLuca DS, Levin JZ, Sivachenko A, Fennell T, Nazaire MD, Williams C, et al. RNA-SeQC: RNA-seq metrics for quality control and process optimization. Bioinformatics. 2012;28:1530–2. 22539670 10.1093/bioinformatics/bts196 PMC3356847

[63] Li B, Dewey CN. RSEM: accurate transcript quantification from RNA-Seq data with or without a reference genome. BMC Bioinforma. 2011;12:323. 10.1186/1471-2105-12-323 PMC3163565 21816040

[64] R Core Team. R: A Language and Environment for Statistical Computing. R Foundation for Statistical Computing, Vienna, Austria 2019.

[65] Bankhead P, Loughrey MB, Fernandez JA, Dombrowski Y, McArt DG, Dunne PD, et al. QuPath: open source software for digital pathology image analysis. Sci Rep. 2017;7:16878. 29203879 10.1038/s41598-017-17204-5 PMC5715110

[66] Schmidt U, Weigert M, Broaddus C, Myers G. Cell detection with star-convex polygons. Springer International Publishing: Cham, 2018, pp 265-73.

[67] Sinha D, Kalimutho M, Bowles J, Chan AL, Merriner DJ, Bain AL, et al. Cep55 overexpression causes male-specific sterility in mice by suppressing Foxo1 nuclear retention through sustained activation of PI3K/Akt signaling. FASEB J. 2018;32:4984–99. 29683733 10.1096/fj.201701096RR

[68] Bacchetti P. Current sample size conventions: flaws, harms, and alternatives. BMC Med. 2010;8:17. 20307281 10.1186/1741-7015-8-17 PMC2856520

[69] Al-Ejeh F, Pajic M, Shi W, Kalimutho M, Miranda M, Nagrial AM, et al. Gemcitabine and CHK1 inhibition potentiate EGFR-directed radioimmunotherapy against pancreatic ductal adenocarcinoma. Clin Cancer Res. 2014;20:3187–97. 24838526 10.1158/1078-0432.CCR-14-0048

