# Correction: CBL0137 and NKG2A blockade: a novel immuno-oncology combination therapy for Myc-overexpressing triple-negative breast cancers

**DOI:** 10.1038/s41388-025-03319-x

**Published:** 2025-03-03

**Authors:** Prahlad V. Raninga, Bijun Zeng, Davide Moi, Ethan Trethowan, Federica Saletta, Pooja Venkat, Chelsea Mayoh, Rochelle C. J. D’Souza, Bryan W. Day, Tyler Shai-Hee, Orazio Vittorio, Roberta Mazzieri, Riccardo Dolcetti, Kum Kum Khanna

**Affiliations:** 1 QIMR Berghofer Medical Research Institute, 300 Herston Road, Herston, Brisbane, QLD 4006 Australia; 2 Mater Research Institute, The University of Queensland, Translational Research Institute, Woolloongabba, QLD 4102 Australia; 3 Peter McCallum Cancer Centre, 305 Grattan Street, Melbourne, VIC 3000 Australia; 4 Sir Peter MacCallum Department of Oncology, The University of Melbourne, Melbourne, VIC Australia; 5 Children’s Cancer Institute, Lowy Cancer Research Centre, UNSW, Kensington, NSW Australia; 6 Murdoch Children’s Research Institute, 50 Flemington Road, Parkville, VIC Australia; 7 School of Clinical Medicine, UNSW Medicine & Health, UNSW Sydney, Kensington, NSW Australia; 8 Department of Microbiology and Immunology, The University of Melbourne, Melbourne, VIC Australia

**Keywords:** Breast cancer, Targeted therapies

Correction to: *Oncogene* 10.1038/s41388-024-03259-y, published online 21 December 2024

The article ‘CBL0137 and NKG2A blockade: a novel immuno-oncology combination therapy for Myc-overexpressing triple-negative breast cancers’, written by Prahlad V. Raninga, Bijun Zeng, Davide Moi, Ethan Trethowan, Federica Saletta, Pooja Venkat, Chelsea Mayoh, Rochelle C. J. D’Souza, Bryan W. Day, Tyler Shai-Hee, Orazio Vittorio5, Roberta Mazzieri, Riccardo Dolcetti and Kum Kum Khanna, was originally published under exclusive license to The Author(s), under exclusive licence to Springer Nature Limited 2024. As a result of the subsequent decision to publish the article under the open access model, the article’s copyright notice was changed on 17. February 2025 to ©The Author(s) 2025 and the article is now distributed under a Creative Commons Attribution 4.0 International License, which permits use, sharing, adaptation, distribution and reproduction in any medium or format, as long as you give appropriate credit to the original author(s) and the source, provide a link to the Creative Commons licence, and indicate if changes were made.

The images or other third party material in this article are included in the article’s Creative Commons licence, unless indicated otherwise in a credit line to the material. If material is not included in the article’s Creative Commons licence and your intended use is not permitted by statutory regulation or exceeds the permitted use, you will need to obtain permission directly from the copyright holder.

To view a copy of this licence, visit http://creativecommons.org/licenses/by/4.0/.

Open Access funding enabled and organized by CAUL and its Member Institutions.

