# Supplementary material for: CBL0137 and NKG2A blockade: a novel immuno-oncology combination therapy for Myc-overexpressing triple-negative breast cancers
Source: Oncogene. 2024 Dec 21;44(13):893–908. doi: 10.1038/s41388-024-03259-y (PMC11932921; doi:10.1038/s41388-024-03259-y)
Supplement: Supplementary file 1 — Supplementary materials and methods [file 41388_2024_3259_MOESM1_ESM.docx]

**Supplementary Materials**

**Methods:**

**MT cell viability assay**

Breast cancer cells were seeded at the density of 3000 cells/well onto a white-walled clear bottom 96-wells plate overnight. Cells were then treated with CBL0137 for 3 days, and cell viability was analyzed using the Real-Time-Glo™ MT Cell Viability Assay (Promega) as per the manufacturer’s guidelines.

**Cell proliferation/growth assay**

Breast cancer cells were seeded at the density of 3000 cells/well onto 96-wells plate overnight. Cells were then treated with CBL0137 for 3 days, and cell viability was analyzed using the CellTiter 96^®^ AQueous One Solution Cell Proliferation Assay (Promega) as per the manufacturer’s guidelines.

**CellTox Green Cytotoxicity assay**

Breast cancer cells were seeded at the density of 3000 cells/well onto a black-walled clear bottom 96-wells plate overnight. Cells were the treated with CBL0137 for 3 days, and cytotoxicity (cell death) was analyzed using the CellTox Green cytotoxicity assay (Promega) as per the manufacturer’s guidelines.

**siRNA and plasmid transfection**

Cells were transfected with 20 nM of non-specific scramble small interfering RNAs (siRNAs), human MYC-specific siRNAs, human SSRP1-specific siRNAs, human p65-specific siRNAs, murine IfngR1-specific, or murine IfngR2-specific siRNAs using Lipofectamine RNAi MAX (Invitrogen Cat #: 13778030) per manufacturer’s instructions. The sequences of siRNAs used in manuscript are provided in Table S5. For plasmid transfection, MDA-MB-361 and MDA-MB-157 cells were transfected with either pcDNA4 empty vector or pcDNA4-MYC plasmid using Lipofectamine 2000 (Promega) as per the manufacturer’s instructions. These constructs were a gift from Professor Wuhan Xiao, Institute of Hydrobiology, Chinese Academy of Sciences.

**Western blot/Immuno blotting**

Breast cancer cells were either transfected with specific siRNAs or MYC overexpression plasmids as described above, or treated with CBL0137 (0-2.5 µM, 24 hours). Proteins were extracted using 7M Urea buffer. Immunoblotting was performed as described previously ^1^ with the antibodies listed in Table S1. The Super Signal chemiluminescent ECL-plus (Amersham) was applied for antibody detection.

**HMGB1 Immunoassay:**

SUM159PT and SUM149PT cells were seeded at the density of 5000 cells/well onto a white-walled clear bottom 96-wells plate overnight. Cells were then treated with CBL0137 for 24 hours, and HMGB1 release in the media was analyzed using the Lumit^TM^ HMGB1 Immunoassay kit (Promega) as per the manufacturer’s guidelines.

SUM159PT and SUM149PT cells were seeded at the density of 5000 cells /well onto a white-walled clear bottom 960wells plate. Cells were reverse transfected with either control or MYC-specific siRNA (20 nM) for 48 hours. HMGB1 expression was then analyzed using the Lumit^TM^ HMGB1 Immunoassay kit (Promega) as per the manufacturer’s guidelines.

**Reverse transcription-quantitative PCR**

Total RNA was extracted using the RNeasy Plus Mini Kit (Qiagen) per manufacturer’s instruction. Reverse transcription quantitative PCR (RT-qPCR) was performed on a CFX384 Touch™ Real-Time PCR Detection System (Bio-Rad) using SYBR™ Green PCR Master Mix (Applied Biosystems, Cat #: 4309155) as described previously ^1^. The list of primers used is listed in Table S2.

**Supplementary tables:**

**Table S1:** **List of the antibodies**

| **Marker** | **Cat #** | **Titration** | **Vendor** |
| --- | --- | --- | --- |
| MYC | ab32072 | 1:1000 | Abcam |
| SSRP1 | 609702 | 1:1000 | BioLegend |
| Total p65 | sc-8008 | 1:500 | Santa Cruz |
| Phospho p65 | 3033T | 1:1000 | Cell Signaling |
| Actin | 612656 | 1:2000 | BD BioSciences |
| Vinculin | 13901S | 1:1000 | Cell Signaling |
| Cyclin A (BF-683) | Sc-239 | 1:500 | Santa Cruz |
| CDK2 (M2) | Sc-163 | 1:500 | Santa Cruz |
| Cyclin B1 (H-433) | Sc-752 | 1:500 | Santa Cruz |
| GAPDH | RDS2275PC100 | 1:1000 | R&D Systems |
| Histone H3 | 4499T | 1:1000 | Cell Signaling |

**Table S2: List of RT-qPCR primers**

| **Gene** | **Forward primer** | **Reverse primer** |
| --- | --- | --- |
| MYC (human) | GCAGCTGCTTAGACGCTGGATTTT | GTTCTCCTCCTCGTCGCAGTAGAAATA |
| MAT2A | CGTGCTGGTAGCCTTGGAGCAA | GATGGGAAGCACAGCACCTCGAT |
| PP1A | CACCGCCGAGGAAAACCGTGTA | GATGGACTTGCCACCAGTGCCAT |
| HK2 | TCGTTCCCCTGCCACCAGACTA | CCCGTGCCCACAATGAGACCAA |
| PGK1 | ACCCAGCTGCTGGGTCTGTCAT | CCACCAGCCTTCTGTGGCAGAT |
| RPL5 | CTGCGCAGCGTATGCACACGAA | GTGAAGGCACCTGGCTGACCAT |
| RPL9 | CCGGGTTGACAAATGGTGGGGTA | GTCTCATCCGAACCCTGCGGAT |
| PYCR1 | GCTTCACAGCAGCAGGCGTCTT | CTGTCCTCAATGTCGGCGCCTA |
| CDK2 | CCTGTGGTACCGAGCTCCTGAA | ACTCCTGGCCACACCACCTCAT |
| RPL32 (human) | CAGGGTTCGTAGAAGATTCAAGGG | CTTGGAGGAAAACATTGTGAGCGATC |
| Myc | GTTGGAAACCCCGCAGACAG | ATAGGGCTGTACGGAGTCGT |
| Oas2 | GGTGGGAGTGTTCACTACAGG | GGGGGTCTGCATTACCTAGAC |
| Oas3 | CAGCGCAGAAGACAAGACCA | TAGACTTCACACAGCGGCCTTT |
| Ifit1 | TACAGCAACCATGGGAGAGAAT | AAGGAACTGGACCTGCTCTG |
| Ifit2 | TCCCTCAAAGCACCAAGTGT | TTCTGTGCAGCACCTCTAAGT |
| Ifih1 | AACAGCGGGAATGAGTCAGG | CGAGTTAGCCAAGTCTGTGT |
| Irf7 | AGCTTGGATCTACTGTGGGC | GGGTTCCTCGTAAACACGGT |
| Il1b | TGCCACCTTTTGACAGTGATG | AAGGTCCACGGGAAAGACAC |
| Clec7a | TGGCGACACAATTCAGGGAG | AGGCTGAGAAAAACCTCCTGTAG |
| Clec12a | CTGTCAGCCCTCTCACCTTTT | AGCATCGGCGGATACTTTTC |
| Ccr2 | GGAGCCATACCTGTAAATGCC | ATGCCGTGGATGAACTGAGG |
| Cxcl9 | CGGACTTCACTCCAACACAGT | TTCCTTATCACTAGGGTTCCTCG |
| H2-T23 (Qa-1b) | CCTTGGAGCTGTGATCATCCT | GGAAGCTCTTGCTGCCTAGAAC |
| IfngR1 | CCGGAGTGGGGAGATCCTAC | ACGGTAAGAGGAGCAACCAC |
| IfngR2 | ACCCAACAGGAACAGGTTGA | GCCCAACGGAATCAGGATGA |
| Rpl32 (Mouse) | TGCCATCTGTTTTACGGCATCA | GATCTGGCCCTTGAACCTTCT |

**Table S3: Reagents for the polyfunctional T cell assay**

| **Marker** | **Clone** | **Fluorochrome** | **Titration** | **Vendor** |
| --- | --- | --- | --- | --- |
| Viability | N/A | FVS440UV | 1/1000 | BD – 566332 |
| Brefeldin A | N/A | N/A | 1/1000 | BioLegend - 420601 |
| CD3 | 17A2 | AF488 | 1/200 | BioLegend - 100210 |
| CD4 | RM4-4 | PE/Cy7 | 1/200 | BioLegend - 116016 |
| CD8 | 53-6.7 | APC/Cy7 | 1/200 | BioLegend - 100714 |
| IFNγ | XMG1.2 | AF700 | 1/00 | BioLegend - 505824 |
| IL2 | JES6-5H4 | PE | 1/100 | BioLegend - 503808 |
| TNFα | MP6-XT22 | AF647 | 1/100 | BioLegend - 506314 |

**Table S4: Reagents for Flow cytometry on tumors**

| **T and NK cell profiling** | | | | |
| --- | --- | --- | --- | --- |
| **Marker** | **Clone** | **Fluorochrome** | **Titration** | **Vendor** |
| Viability | N/A | FVS440UV | 1/1000 | BD – 566332 |
| CD45 | 30-F11 | BUV563 | 1/800 | BD – 565710 |
| CD3 | 145-2C11 | BUV737 | 1/200 | BD – 564618 |
| CD4 | GK1.5 | BUV496 | 1/400 | BD – 564667 |
| CD8 | 5.3-6.7 | BUV805 | 1/400 | BD – 564920 |
| FoxP3 | FJK-16s | PE-Cy5 | 1/200 | eBio – 15-5773-82 |
| CD49b | HMα2 | BV786 | 1/400 | BD – *OptiBuild* |
| CD44 | IM7 | APC-Cy7 | 1/400 | BD – 560568 |
| CD279 (PD-1) | J43 | BUV395 | 1/100 | BD - *OptiBuild* |
| Granzyme B | QA16A02 | PE-Dazzle594 | 1/400 | BL – 372216 |
| CD69 | H1.2F3 | BV480 | 1/100 | BD – *OptiBuild* |
| CD226 (DNAM-1) | TX42.1 | BV650 | 1/200 | BL – 133621 |
| CD314 (NKG2D) | CX5 | BV711 | 1/200 | BD – 563694 |
| CD152 (CTLA-4) | UC10-4F10-11 | APC-R700 | 1/200 | BD – 565778 |
| CD366 (TIM-3) | RMT3-23 | FITC | 1/400 | eBio – 11-5870-82 |
| TIGIT | 1G9 | BV421 | 1/200 | BD – 565270 |
| CD223 (LAG-3) | C9B7W | BV750 | 1/200 | BD – *OptiBuild* |
| VISTA | MIH64 | PE | 1/200 | BD – 566270 |
| NKG2A/C/E | 20d5 | BV605 | 1/200 | BD – 564382 |
| Eomes | Dan11mag | PE-Cy7 | 1/200 | eBio – 25-4875-82 |
| T-bet | 4B10 | AF647 | 1/400 | BL – 644804 |

| **B cell profiling** | | | | |
| --- | --- | --- | --- | --- |
| **Marker** | **Clone** | **Fluorochrome** | **Titration** | **Vendor** |
| Viability | N/A | FVS440UV | 1/1000 | BD-566332 |
| CD45 | 30-F11 | BUV563 | 1/800 | BD-612924 |
| CD49b | HMα2 | BV786 | 1/400 | BD-*740895* |
| CD11b | M1/70 | BUV661 | 1/800 | BD-612977 |
| CD19 | ID3 | BUV395 | 1/400 | BD-563557 |
| CD45R (B220) | RA3-6B2 | BB700 | 1/400 | BD-746206 |
| IgM | R6-60.2 | PE-Cy7 | 1/800 | BD-552867 |
| MHC II (I-A/I-E) | 2G9 | BV605 | 1/800 | BD-743872 |

| **Myeloid cell profiling** | | | | |
| --- | --- | --- | --- | --- |
| **Marker** | **Clone** | **Fluorochrome** | **Titration** | **Vendor** |
| Viability | N/A | FVS440UV | 1/1000 | BD-566332 |
| CD45 | 30-F11 | BUV563 | 1/800 | BD-612924 |
| CD11b | M1/70 | BB515 | 1/800 | BD-564454 |
| F480 | BM8 | BV510 | 1/100 | BD-123135 |
| Ly6G | 1A8 | BUV805 | 1/400 | BD-741994 |
| Ly6C | HK1.4.rMAb | BUV737 | 1/400 | BD-755201 |
| CD80 | 16-10A1 | APC-Fire 750 | 1/200 | BL-104740 |
| CD274 (PDL1) | 10F.9G2 | RealBlue 780 | 1/800 | BD-569146 |

**Table S5: List of the siRNAs.**

| **Gene** | **siRNA Sequence (Sense)** | **siRNA Sequence (Anti-sense)** |
| --- | --- | --- |
| MYC | AUCAUUGAGCCAAAUCUUAAAAAAA | UAUAGUAACUCGGUUUAGAAUUUUUUU |
| SSRP1 | GUACCAGUUUCUCCUCAUGAAAUGC | ACCAUGGUCAAAGAGGAGUACUUUACG |
| P65 | CAUGGAUUCAUUACAGCUUAAUCAA | UUGAUUAAGCUGUAAUGAAUCCAUGAU |
| IfngR1 | CAGACUUAGAUACGUGAAUAAGGAT | UAGUCUGAAUCUAUGCACUUAUUCCUA |
| IfngR2 | AGAGAGGUGAUUAAUCUUGUAAATA | UGUCUCUCCACUAAUUAGAACAUUUAU |

**Table S6:** **List of the antibodies used for Opal multi-plexed immunohistochemistry.**

| Antibody | Cat # | Antibody dilution | Opal fluorophore pair | Opal dilution | Vendor |
| --- | --- | --- | --- | --- | --- |
| CD4 | ab183685 | 1:1250 | Opal 480 | 1:300 | Abcam |
| CD8 | ab209775 | 1:1000 | Opal 570 | 1:150 | Abcam |
| CD25 | ab227834 | 1:1000 | Opal 620 | 1:150 | Abcam |
| NCR1 | ab233558 | 1:1000 | Opal 690 | 1:150 | Abcam |
| FoxP3 | ab215206 | 1:1250 | Opal 520 | 1:150 | Abcam |
| CD11b | ab133357 | 1:40000 | Opal 780 | 1:25 | Abcam |

1 Raninga PV, Lee A, Sinha D, Dong LF, Datta KK, Lu X *et al*. Marizomib suppresses triple-negative breast cancer via proteasome and oxidative phosphorylation inhibition. Theranostics 2020; 10: 5259-5275.

**SUPPLEMENTARY FIGURES:**

***Figure S1:***


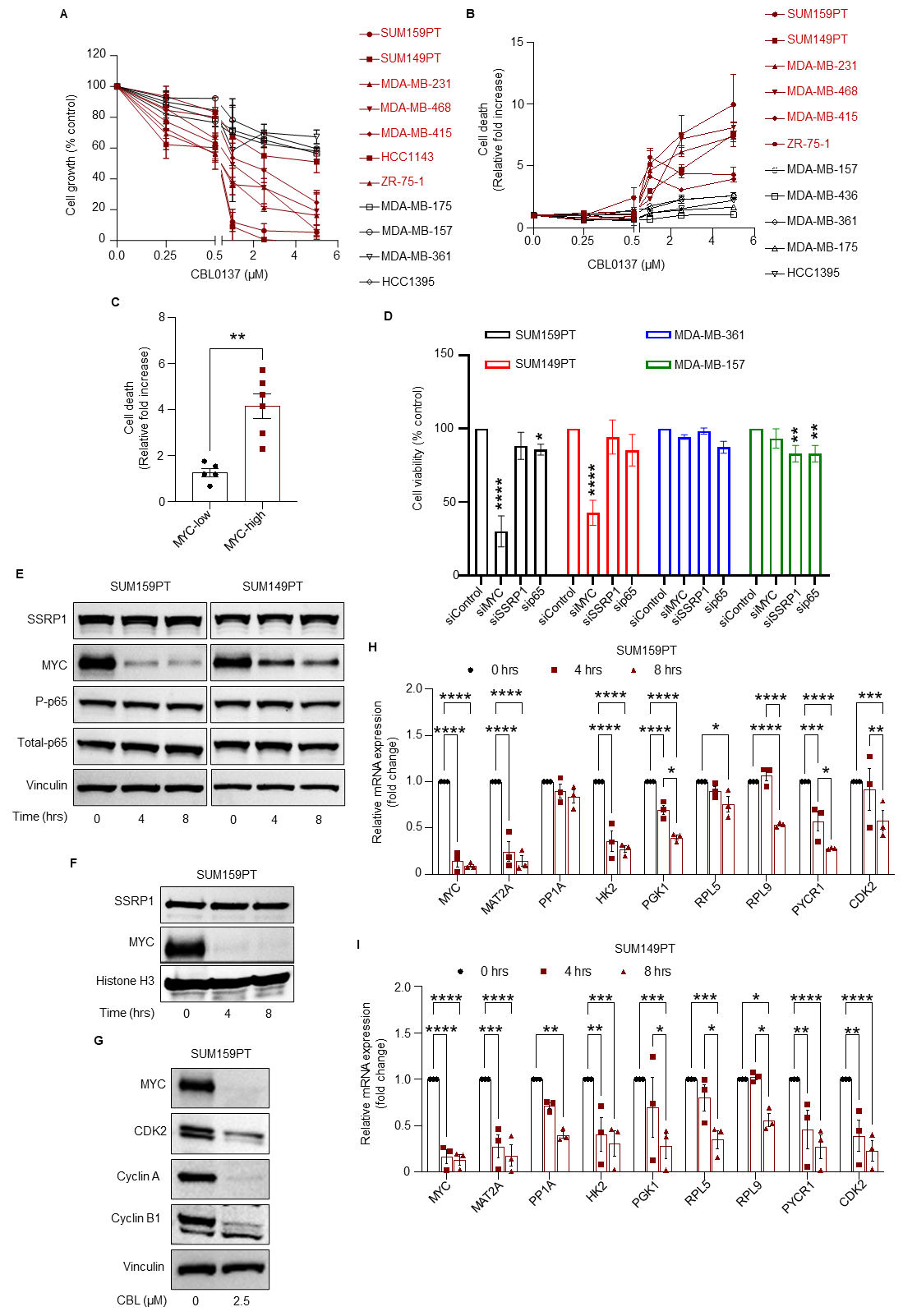


***Figure S1: CBL0137 inhibits proliferation in MYC-high breast cancer cells via MYC pathway inhibition.***

**(A)** A panel of breast cancer cell lines were treated with CBL0137 (0-5 µM) for 72 hours. Cell viability was assessed by MT cell viability assays and the growth curve for each MYC-high and MYC-low breast cancer cell line is shown. Data are presented as mean ± SEM (n=3).

**(B)** A panel of breast cancer cell lines were treated with CBL0137 (0-5 µM) for 72 hours. Cell death was assessed by CellTox Green cytotoxicity assays and increase in cell death in each MYC-high and MYC-low breast cancer cell line is shown. Data are presented as mean ± SEM (n=3).

**(C)** Mean fold change in cell death in MYC-low and MYC-high breast cancer cell lines upon CBL0137 treatment (5 µM) assessed by CellTox Green cytotoxicity assays. Data are presented as mean ± SEM (n=3). t-Test, **p<0.01

**(D)** Two MYC-high (SUM159PT and SUM149PT) and two MYC-low (MDA-MB-361 and MDA-MB-157) TNBC cell lines were transfected either with MYC-specific siRNA, SSRP1-specific siRNA, p65-specific siRNAs or scramble/control siRNAs. Cell viability was assessed by trypan blue cell viability assays 6 days post-transfection. Data are presented as mean ± SD (n=3). Two-way ANOVA with Tukey’s multiple comparisons test, *p<0.05, **p<0.01, ***p<0.001, ****p<0.0001.

**(E)** SUM159PT and SUM149PT cells were treated with 2.5 µM CBL0137 for indicated time period. Protein was harvested and MYC, phospho-p65, total-p65, and SSRP1 protein levels were analysed by Western blot analysis. Vinculin was used as a loading control. Images are representative of three independent experiments.

**(F)** SUM159PT cells were treated with 2.5 µM CBL0137 for indicated time period. Chromatin-bound proteins were harvested by sub-cellular fractionation, and MYC and SSRP1 protein levels were analysed by Western blot analysis. Histone H3 was used as a loading control. Images are representative of three independent experiments.

**(G)** SUM159PT cells were treated with 2.5 µM CBL0137 for 8 hours. Protein levels of MYC and its downstream targets (CDK2, Cyclin A, and Cyclin B1) were analysed by Western blot analysis. Vinculin was used as a loading control.

**(H, I)** SUM159PT (H) and SUM149PT (I) cells were treated with 2.5 µM CBL0137 for indicated time period. mRNA levels of MYC and its indicated transcriptional target genes were analysed by RT-qPCR. Data are presented as mean ± SEM (n=3); Two-way ANOVA with Tukey’s multiple comparisons test, *p<0.05, **p<0.01, ***p<0.001, ****p<0.0001.

***Figure S2:***


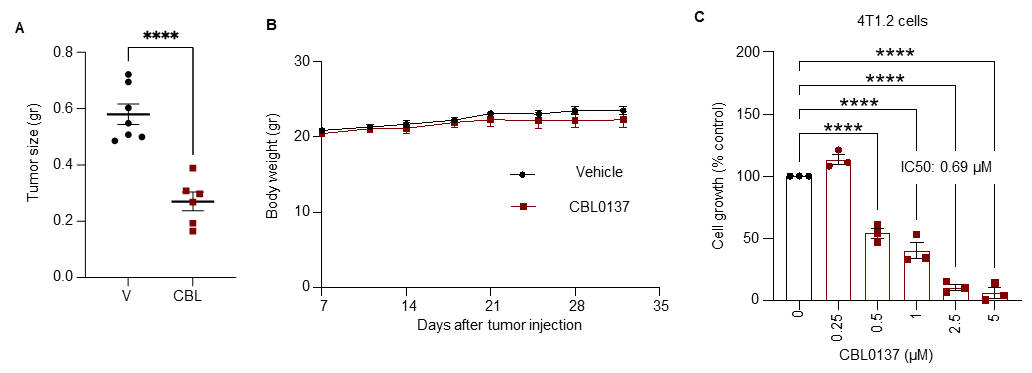


***Figure S2: In vivo anti-cancer activity of CBL0137 in the MDA-MB-231 model.***

**(A)** Tumor size/weight (grams) in NSG mice orthotopically injected with MDA-MB-231 cells following treatment with vehicle or CBL0137 (60 mg/kg, once/week, i.v.) for three weeks. Tumour weight were measured at the end of the treatment. Data are presented as mean ± SEM (n=7 mice/group); t-Test, ****p<0.0001

**(B)** Body weight of NSG mice orthotopically injected with MDA-MB-231 cells following treatment with vehicle or CBL0137 (60 mg/kg, once/week, i.v.) for three weeks. Data are presented as mean ± SEM (n=7 mice/group).

**(C)** Murine 4T1.2 breast cancer cells were treated *in vitro* with CBL0137 (0-5 µM) for 72 hours. Cell growth was analyzed using MTS cell proliferation assay. Data are presented as mean ± SEM (n=3 technical replicates); One-way ANOVA with Tukey’s multiple comparisons test, ****p<0.0001.

***Figure S3:***


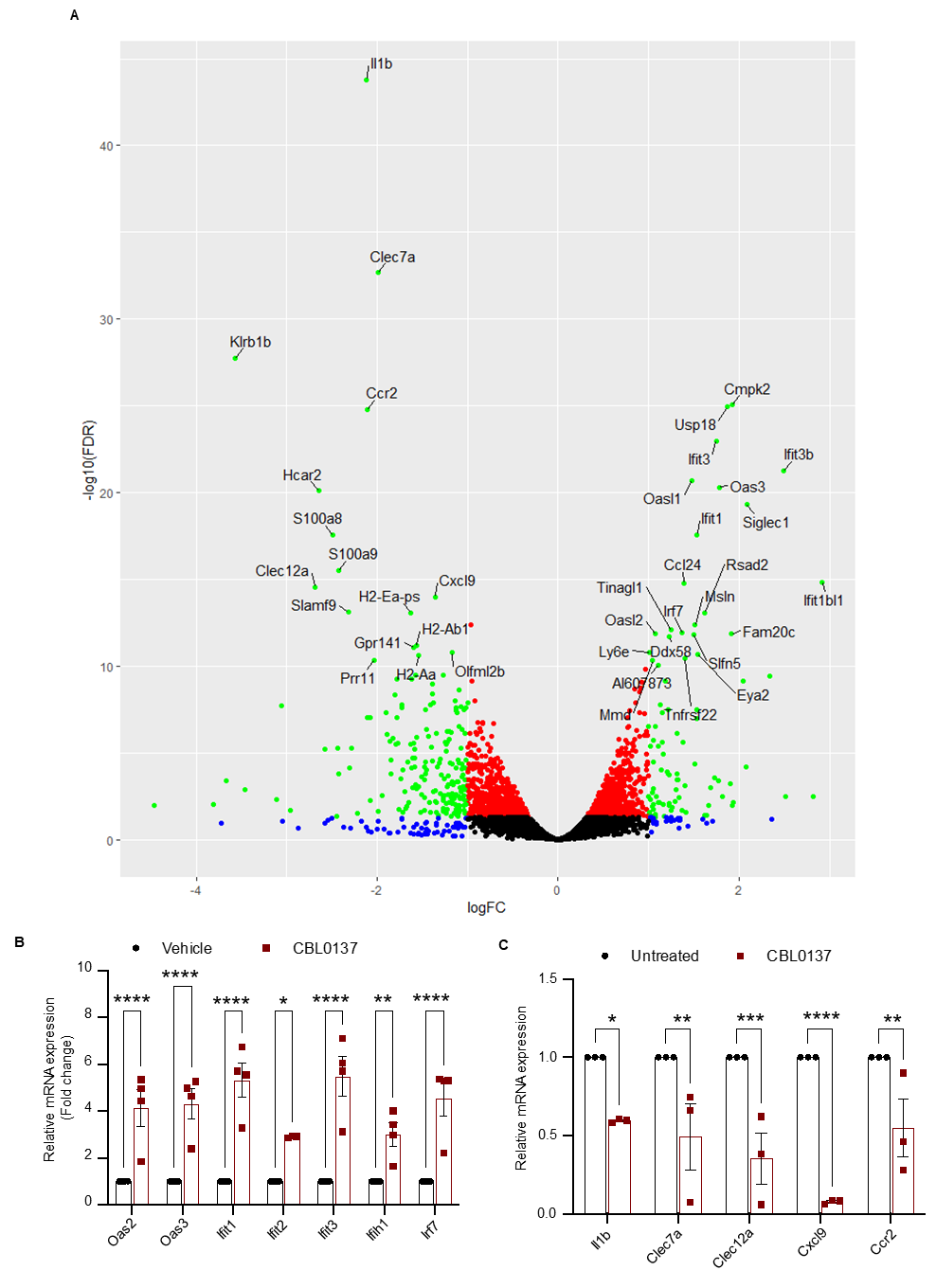


***Figure S3: Differentially expressed gene upon CBL0137 treatment in 4T1.2 tumors in vivo.***

Murine 4T1.2 tumor-bearing Balb/c mice were treated with vehicle or CBL0137 (60 mg/kg, IV) for one week. Tumors from vehicle-treated and CBL0137-treated mice were collected for whole transcriptome analysis by RNA sequencing.

**(A)** Volcano plot showing the differentially expressed genes (both up- and down-regulated) upon CBL0137 treatment compared to vehicle treatment. Genes in red with FDR<0.05, genes in blue Fold Change (FC)>2, genes in green with FDR<0.05 & FC>2.

**(B)** mRNA levels (RT-qPCR) of IFNγ pathway genes (Oas2, Oas3, Ifit1, Ifit2, Ifit3, Ifih1, and Irf7) in 4T1.2 tumors treated with vehicle or CBL0137 (60 mg/kg) for one week. Data are presented as mean ± SEM (n=4 tumor/group); Two-way ANOVA followed by Sidak’s post-test, *p<0.05, ***p<0.001, ****p<0.0001

**(C)** mRNA levels (RT-qPCR) of genes encoding cytokines (IL-1b), chemokine (Ccr2) as well as cell surface receptor known to play a role in innate immunity (Clec12a) in 4T1.2 tumors treated with vehicle or CBL0137 (60 mg/kg) for one week. Data are presented as mean ± SEM (n=4 tumor/group); t-Test, *p<0.05, ****p<0.0001

***Figure S4:***


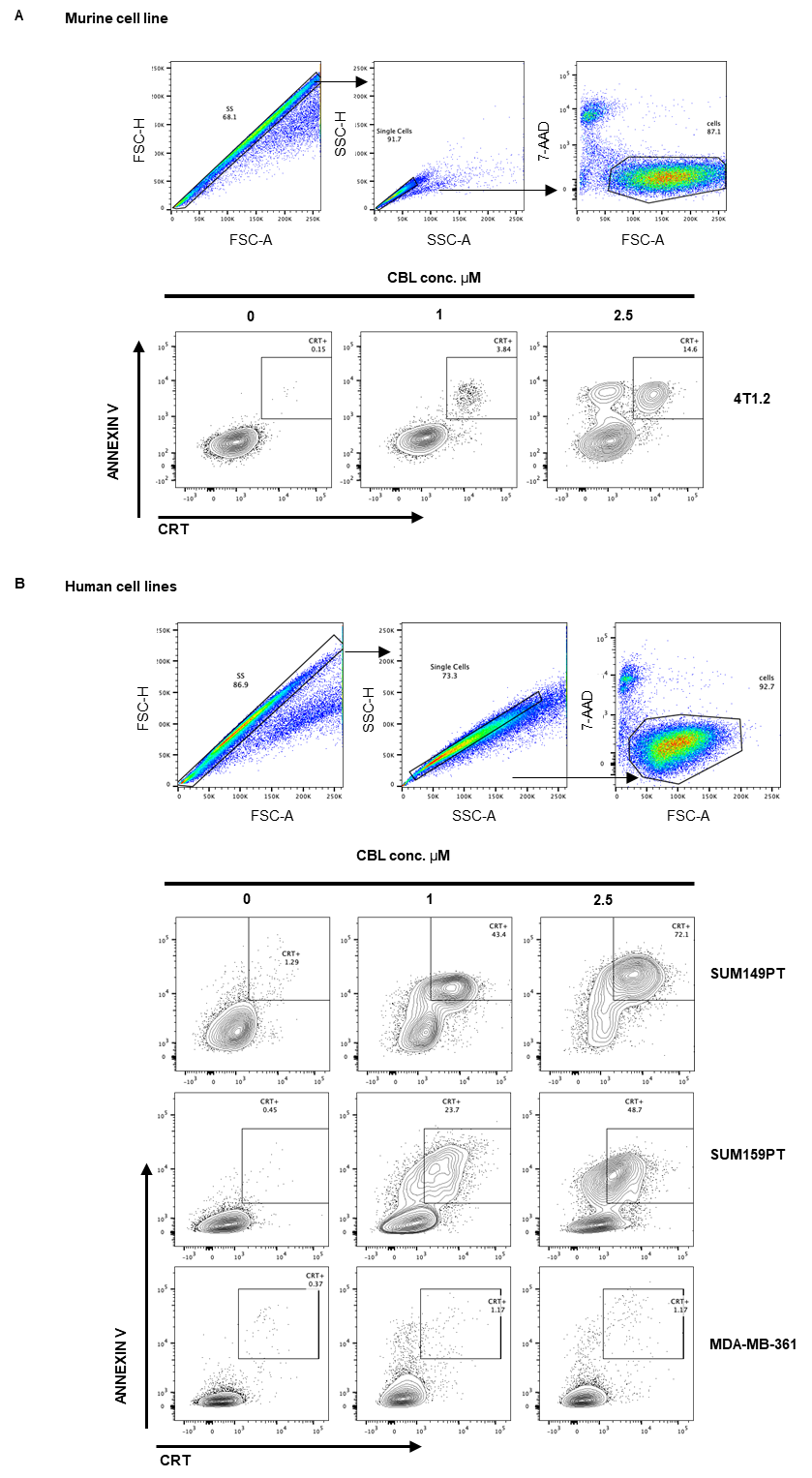


***Figure S4: Gating strategy related to Figure 3A-D.***

Physical parameters and 7-AAD positivity were used to exclude doublets and late apoptotic cells. Single cells including live cells and early apoptotic cells were then analyzed to quantity the percentages of CRT exposure on early apoptotic cells (Annexin V^+^).

**(A)** Representative dot blots for the murine 4T1.2 cell line.

**(B)** Representative dot blots for the indicated human cell lines.

***Figure S5:***


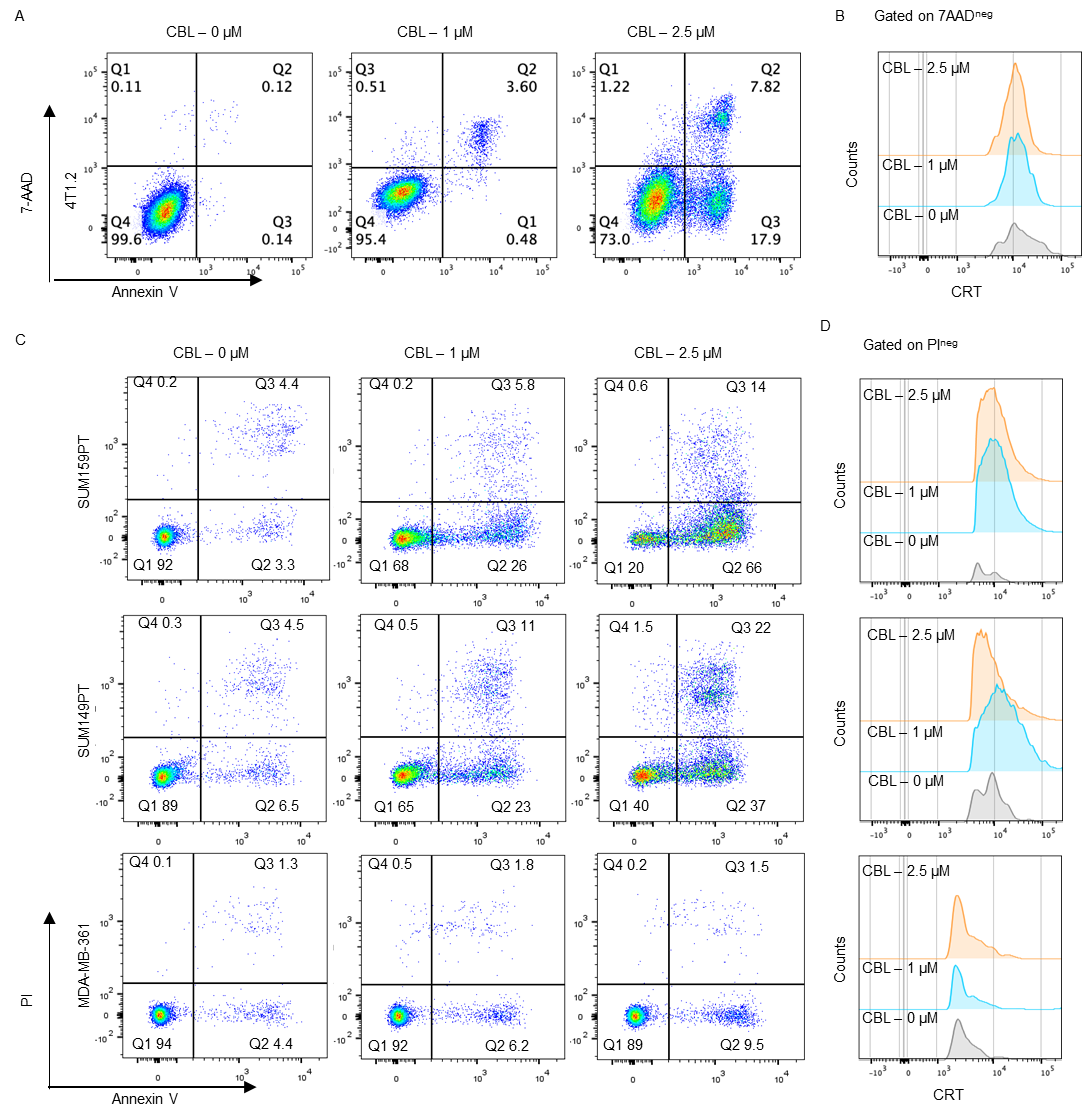


***Figure S5: Gating strategy related to Figure 3A-D***

**(A, C)** Representative dot blots for the identification of live (double negative), early apoptotic (Annexin V^+^ 7-AAD^-^ or PI^-^), and late apoptotic (double positive) cells in murine 4T1.2 (A) or the indicated human cell lines (C) treated with 0, 1, or 2.5 µM CBL0137. Analysis was performed on total single cells.

**(B, D)** Representative histograms for the visualization of CRT exposure in in murine 4T1.2 cells (B) or the indicated human cell lines (D) treated with 0, 1, or 2.5 µM CBL0137. Analysis was performed on 7AAD^neg^ (B) or PI^neg^ (D) cells.

***Figure S6:***


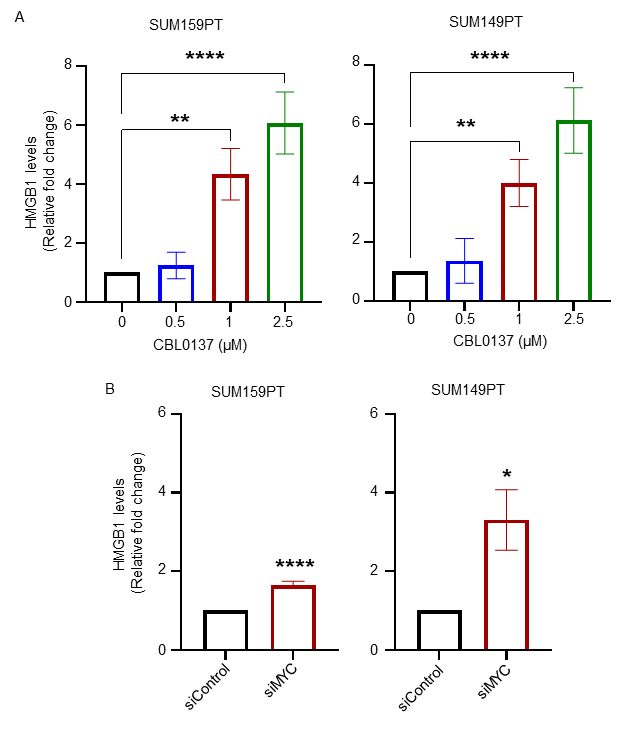


***Figure S6. Effect of CBL0137 treatment and MYC knockdown on HMGB1 levels.***

**(A)** Two MYC-high TNBC cell lines, SUM159PT and SUM149PT, were treated with CBL0137 (0-2.5 µM) for 24 hours, and HMGB1 levels was analysed in the media by using HMGB1 Immunoassay kit (Promega). Data are presented as mean ± SEM; One-way ANOVA followed by Tukey’s post-test, **p<0.01, ****p<0.0001.

**(B)** SUM159PT and SUM149PT cells were transfected with either control or MYC-specific siRNA for 48 hours. HMGB1 levels in the media were analysed 48 hours post-transfection using HMGB1 Immunoassay kit (Promega). Data are presented as mean ± SEM; t-Test, *p<0.05, ****p<0.0001.

***Figure S7:***


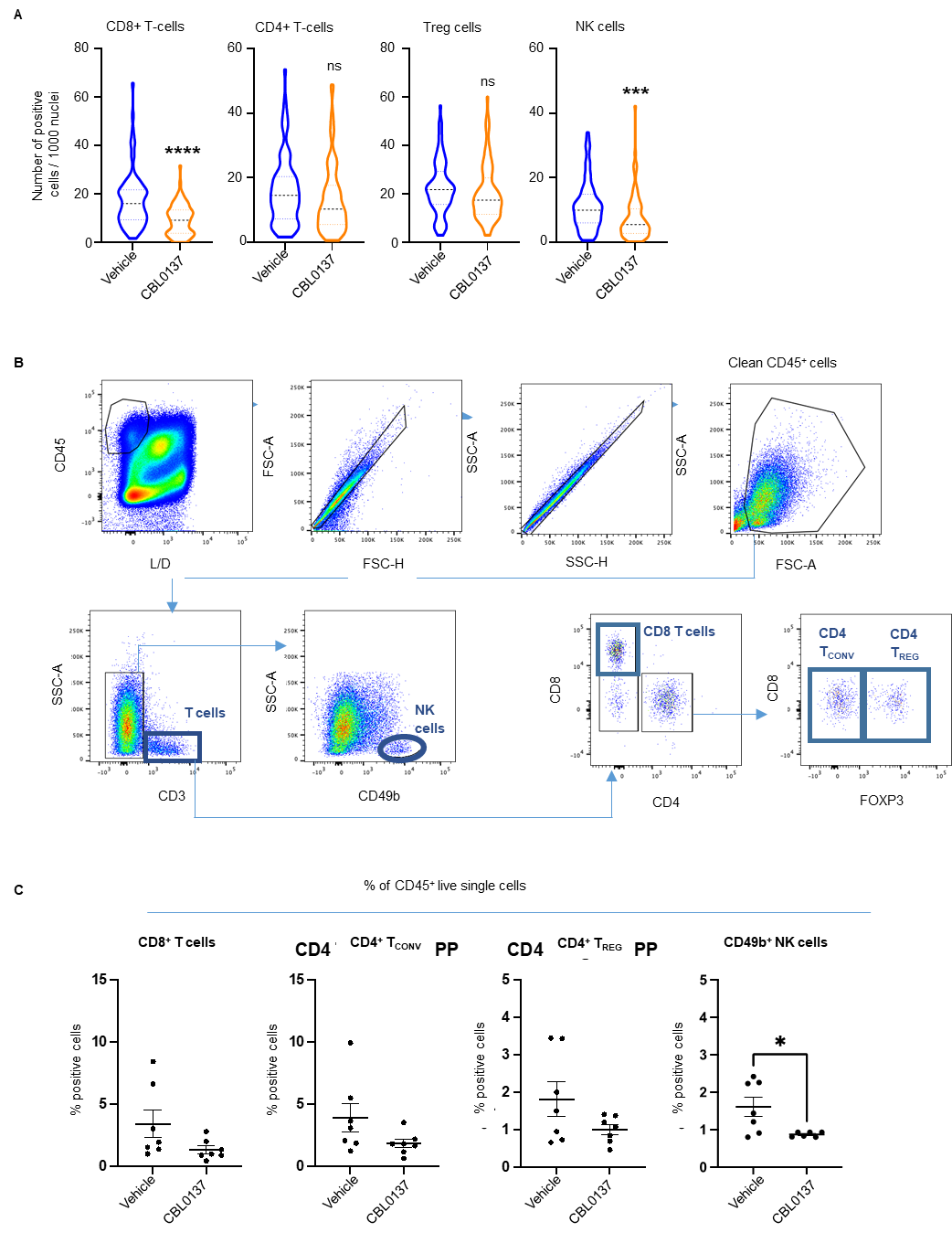


***Figure S7. Immune profiling of T and NK cells in 4T1.2 tumors treated with CBL0137***

Mice and tumors as described in Figure 4A.

***(A)*** Multiplexed immunofluorescence staining of vehicle- and CBL0137-treated (60 mg/kg) 4T1.2 mouse tumors. Six mice were analysed under each condition. Tumor from each mouse was subjected to Opal staining: CD8^+^ T cells, NK cells (marked by NCR1^+^ cells), CD4^+^ T cells, and Treg (CD4^+^ CD25^+^ FoxP3^+^) cells. A comparative summary of the quantification and relative significance is presented. Data are presented as mean ± SEM; t-Test, ***p<0.001, ****p<0.0001.

***(B***) Gating strategy for the flow cytometric analysis presented in Figure 5, 6 and S8-S11. Physical parameters, L/D positivity and CD45 positivity were used to exclude dead cells, doublets, and debris, and to identify live tumor infiltrating immune cells. CD4^+^ and CD8^+^ T cells were gated on CD3^+^ cells and NK cells were identified within CD3^neg^ fraction. CD4^+^ T cells were further divided into T_conv_ (FoxP3^neg^) and T_reg_ (FoxP3^+^) cells.

**(C)** Percentages CD8^+^ T cells, CD4^+^ T_conv_, CD4^+^ T_reg_, and NK cells within CD45^+^ live single cells. Data are presented as mean ± SEM; t-Test, *p<0.05.

***Figure S8:***


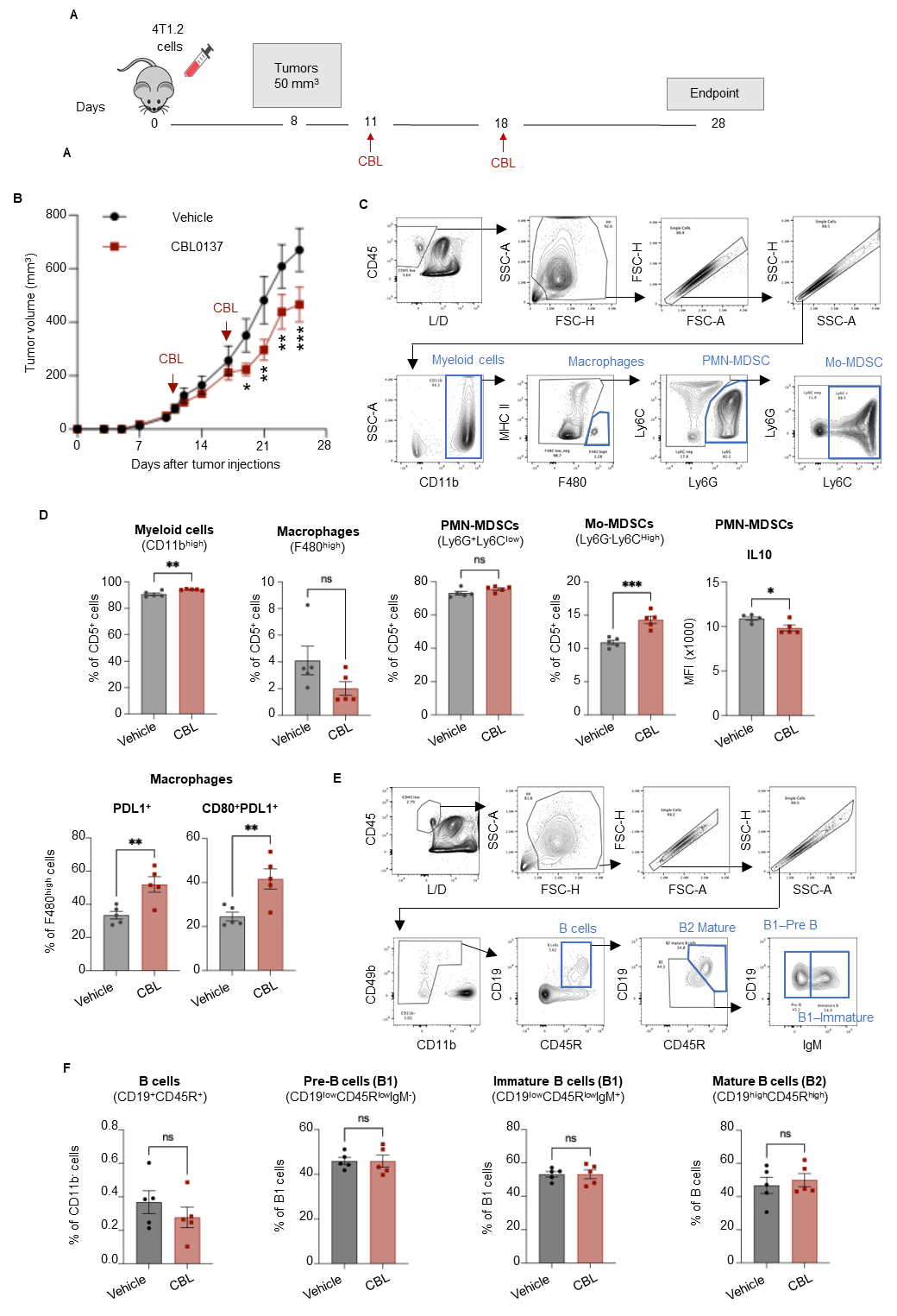


***Figure S8: Immune profiling of myeloid and B cells in 4T1.2 tumors treated with CBL0137***

**(A)** Experimental scheme: BAlb/c mice were orthotopically injected with 4T1.2 cells and 2 weekly treatments with CBL0137 (60 mg/kg) were started after the tumors reached a size of at least 50 mm3. Control mice received vehicle only. Tumors were collected on day 25 for immune profiling by flow cytometry.

**(B)** Tumor growth over time measured by caliper. Data are presented as mean ± SEM (n=5 mice/group); 2-way-Anova, *p<0.05, **p<0.01, ***p<0.001.

**(C)** Gating strategy for the flow cytometric analysis presented in (D). L/D positivity, CD45 positivity and physical parameters, were used to exclude dead cells, doublets, and debris, and to identify live tumor infiltrating immune cells. Myeloid cells (CD11b^high^) were gated on single live CD45^+^ cells. Macrophages (F480^high^) were gated on CD11b^high^ cells. Polymorphonuclear myeloid-derived suppressor cells (PNM-MDSCs, Ly6G^+^Ly6C^low^) were gated on F480^neg^ cells. Monocytic myeloid-derived suppressor cells (Mo-MDSCs, Ly6G^neg^Ly6C^high^) were gated on Ly6G^neg^ cells.

**(D)** Left panel: percentages Myeloid cells, Macrophages, PNM-MDSCs, and Mo-MDSCs within CD45^+^ cells. Middle panel: percentages PDL1^+^ and CD80^+^PDL1^+^ Macrophages. Right panel: IL10 expression levels in PMN-MDSCs. Data are presented as mean ± SEM; t-Test, ns = non-significant, *p<0.05, **p<0.01, **p<0.001.

**(E)** Gating strategy for the flow cytometric analysis presented in (F). L/D positivity, CD45 positivity and physical parameters, were used to exclude dead cells, doublets, and debris, and to identify live tumor infiltrating immune cells. B cells (CD19^+^CD45R^+^) were gated on CD11b^-^ cells. B1 (CD19^low^CD45R^low^) and B2-mature (CD19^high^CD45R^high^) cells were gated on total B cells. B1 cells were further divided into B1-Pre (IgM^-^) and B1-immature (IgM^+^).

**(F)** Percentages total B, B1-Pre, B1-immature and B2-mature cells within the indicated populations. Data are presented as mean ± SEM; t-Test, ns = non-significant.

***Figure S9:***


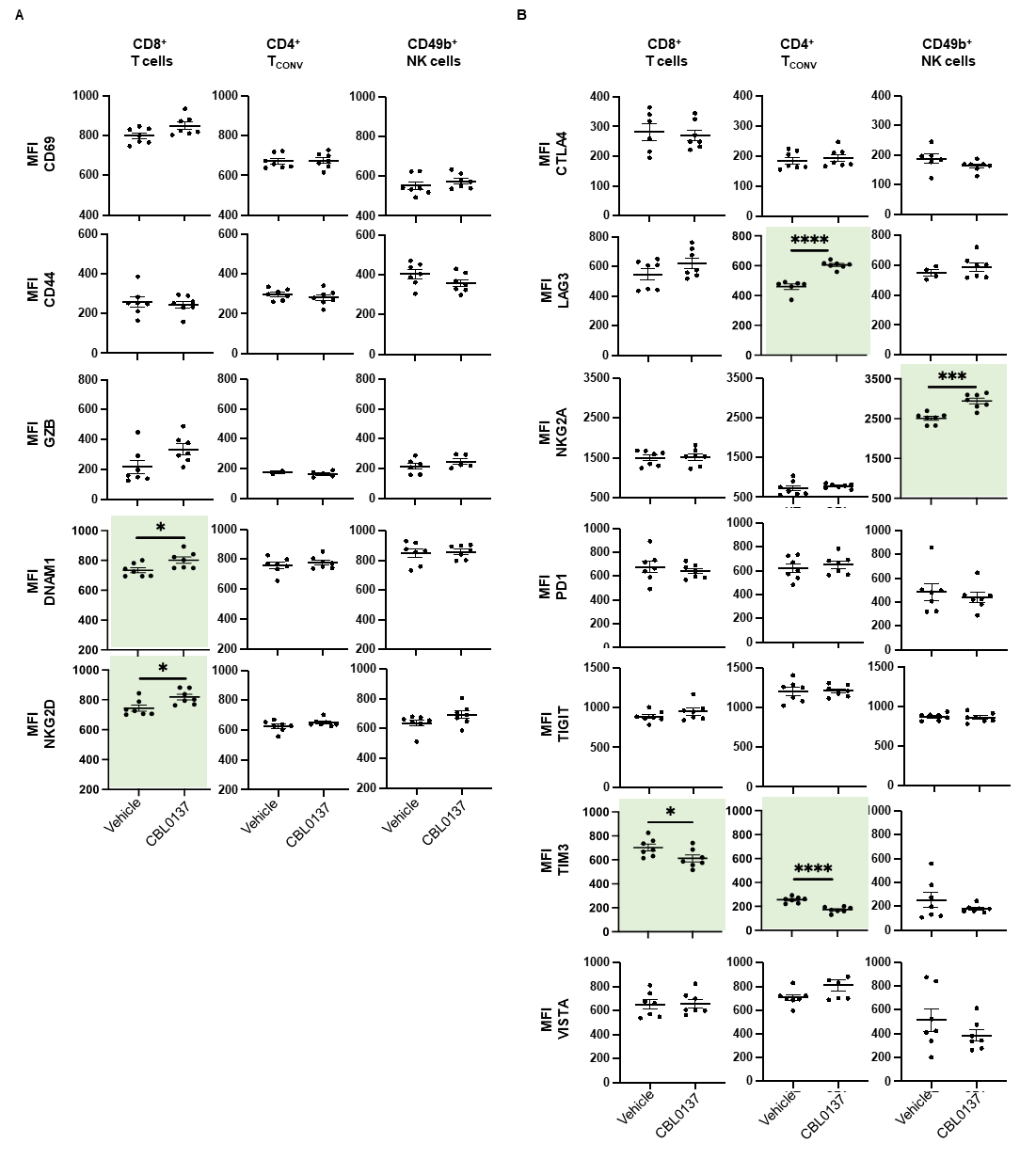


***Figure S9: Expression levels of the markers presented in Figure 5A & 5B.***

***(A, B)*** Mice and tumors as described in Figure 4A. Mean fluorescent intensity of the indicated activation markers (A) or inhibitory checkpoint molecules (B) in CD8^+^ T cells (left), CD4^+^ T_conv_ cells (centre) and NK cells (right) are presented as mean ± SEM. A green background highlights significantly different MFIs; t-Test, *p<0.05, ***p<0.001, ****p<0.0001.

***Figure S10:***


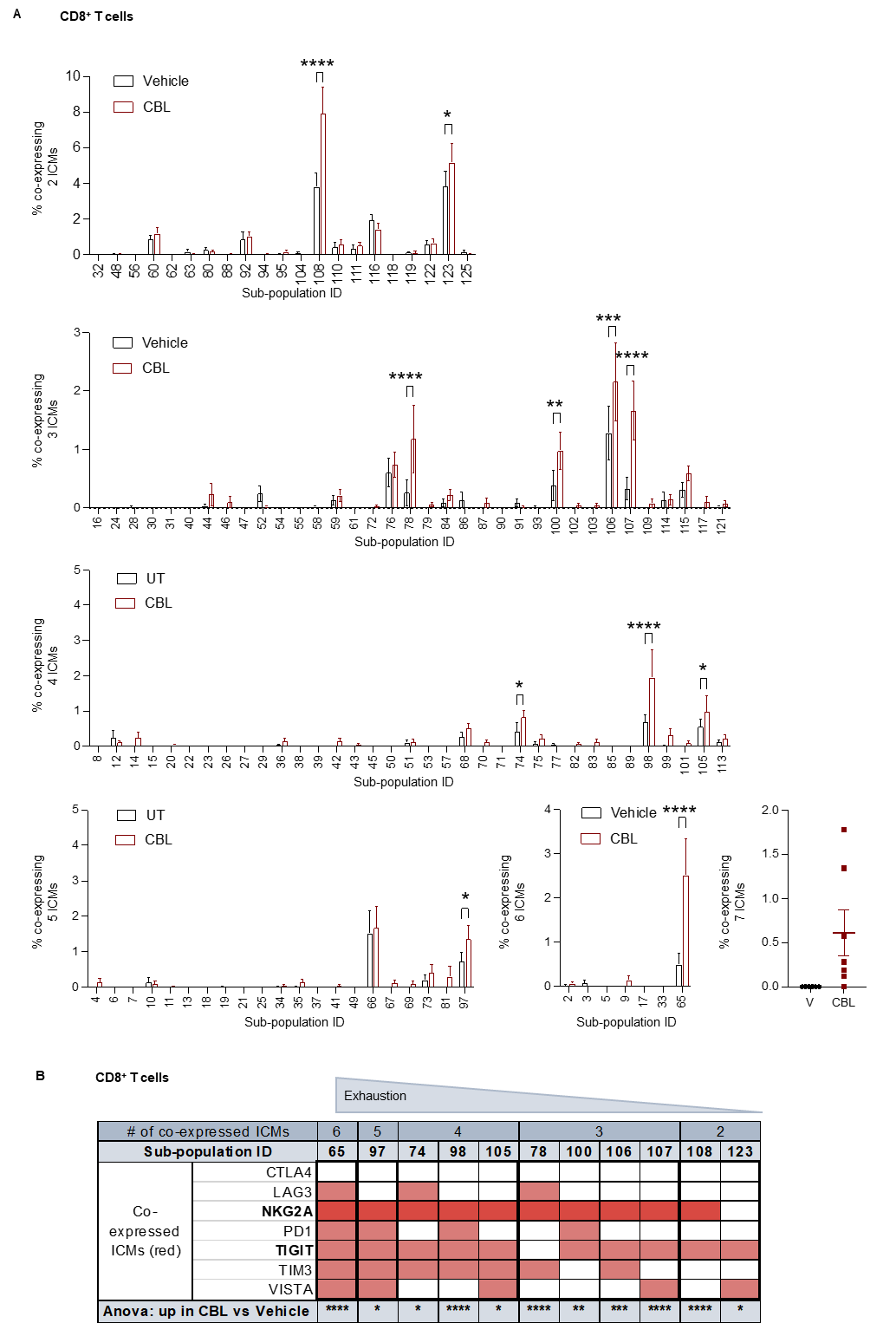


***Figure S10: Boolean gating analysis in CD8+ T cells***

Mice and tumors as described in Figure 4A.

**(A)** Boolean gating analysis for the co-expression of the indicated number of inhibitory checkpoint molecules (ICMs). Each graph reports the percentages of cell sub-populations expressing all possible combinations of the indicated numbers of (ICMs). Each combination of ICMs defines a specific sub-population identified by a progressive number (Sub-population ID). Data are presented as mean ± SEM; Two-way ANOVA with Fisher’s LSD multiple comparisons test, *p<0.05, **p<0.01, ***p<0.001, ****p<0.0001.

**(B)** Table summarizing the specific ICM combination in each subpopulation significantly expanded (up) in tumors from CBL0137 treated mice when compared with control tumors.

***Figure S11:***


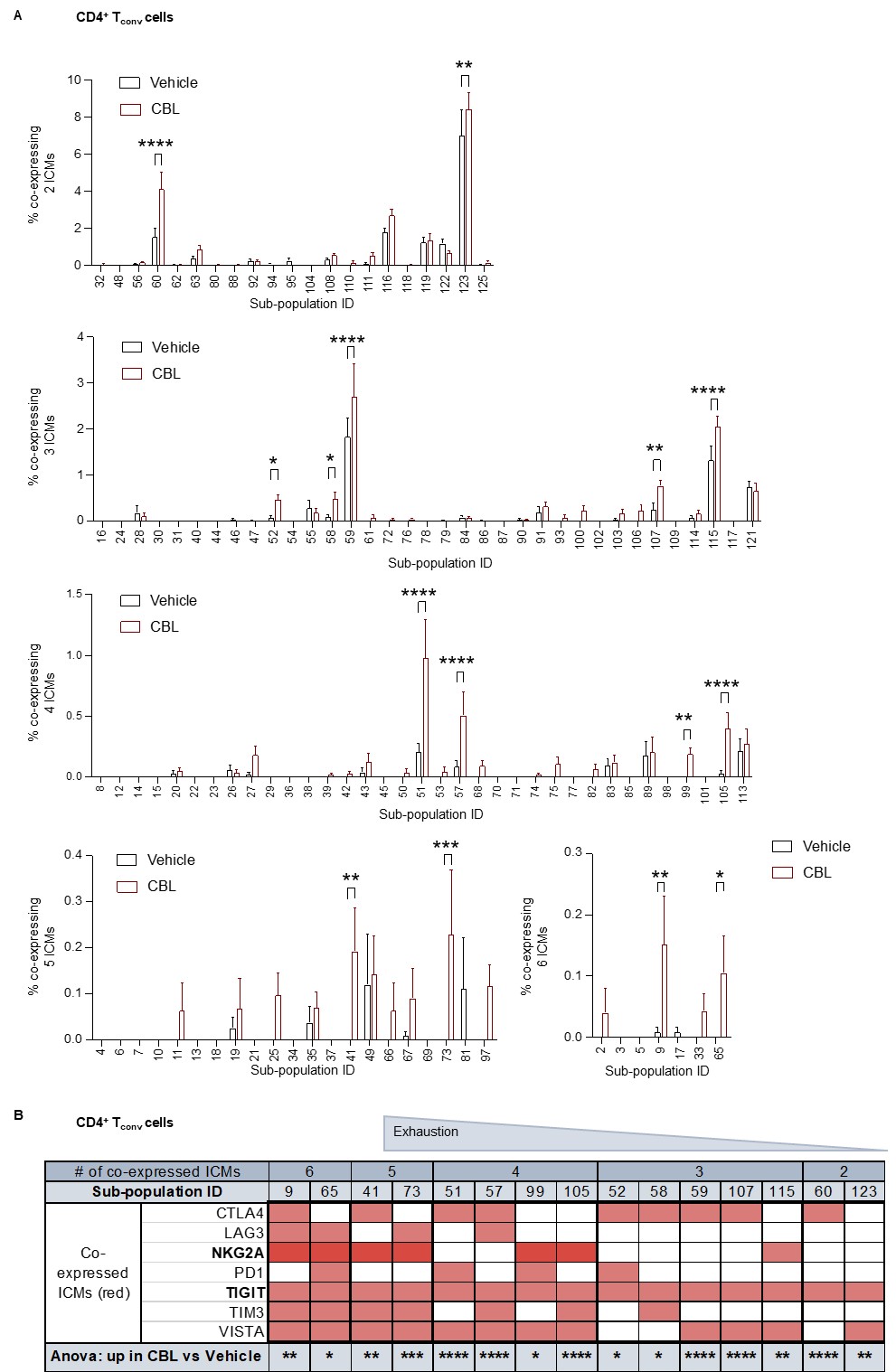


***Figure S11: Boolean gating analysis in CD4+ T_conv_ cells***

Mice and tumors as described in Figure 4A.

**(A)** Boolean gating analysis for the co-expression of the indicated number of inhibitory checkpoint molecules (ICMs). Each graph reports the percentages of cell sub-populations expressing all possible combinations of the indicated numbers of (ICMs). Each combination of ICMs defines a specific sub-population identified by a progressive number (Sub-population ID). Data are presented as mean ± SEM; Two-way ANOVA with Fisher’s LSD multiple comparisons test, *p<0.05, **p<0.01, ***p<0.001, ****p<0.0001.

**(B)** Table summarizing the specific ICM combination in each subpopulation significantly expanded (up) in tumors from CBL0137 treated mice when compared with control tumors.

***Figure S12:***


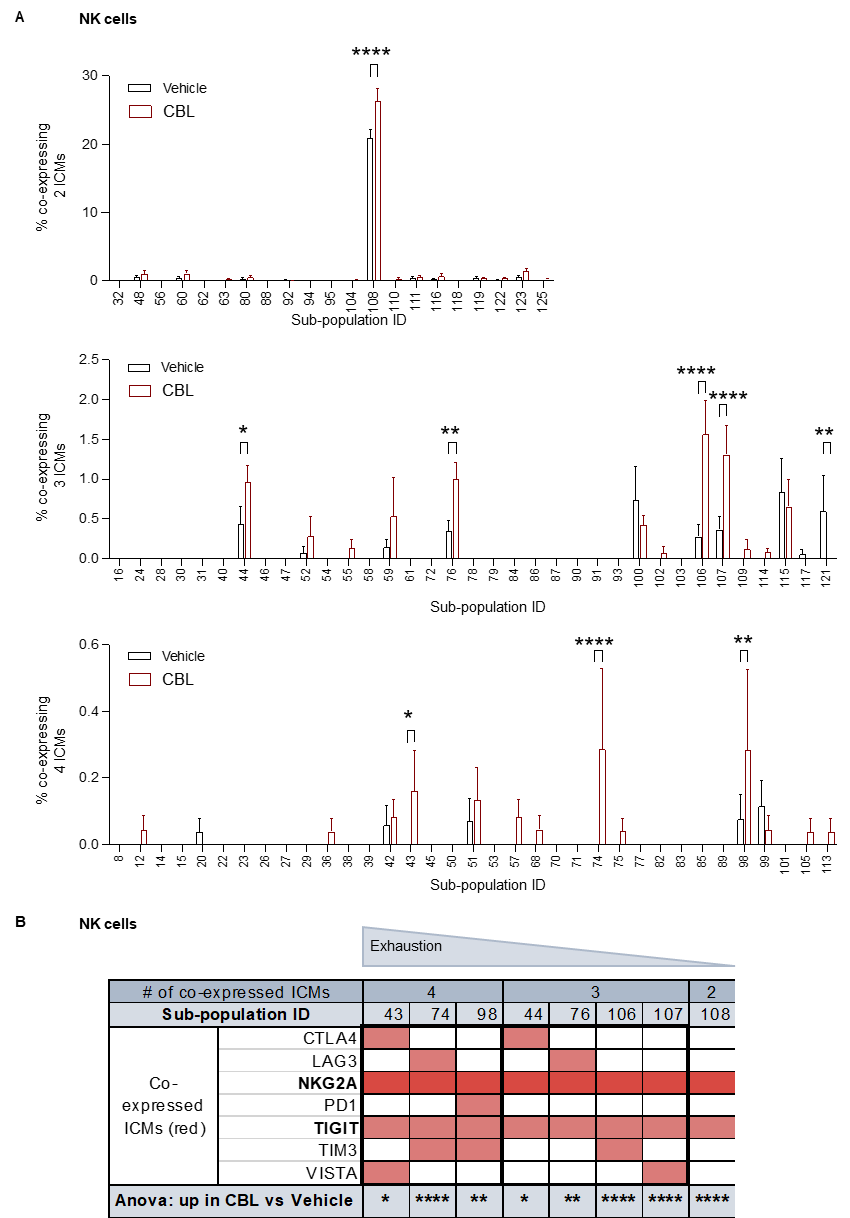


***Figure S12: Boolean gating analysis in NK cells***

Mice and tumors as described in Figure 4A.

**(A)** Boolean gating analysis for the co-expression of the indicated number of inhibitory checkpoint molecules (ICMs). Each graph reports the percentages of cell sub-populations expressing all possible combinations of the indicated numbers of (ICMs). Each combination of ICMs defines a specific sub-population identified by a progressive number (Sub-population ID). Data are presented as mean ± SEM; Two-way ANOVA with Fisher’s LSD multiple comparisons test, *p<0.05, **p<0.01, ***p<0.001, ****p<0.0001.

**(B)** Table summarizing the specific ICM combination in each subpopulation significantly expanded (up) in tumors from CBL0137 treated mice when compared with control tumors.

***Figure S13:***


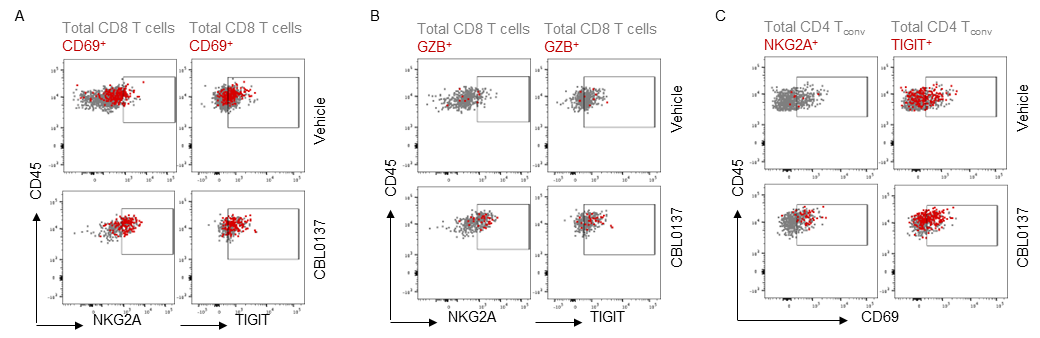


***Figure S13: Back gating analysis related to figure 6A***

Mice and tumors as described in Figure 4A.

**(A)** CD8^+^ T cells expressing CD69 (red) were backgated on total CD8^+^ T cells (gray) and analyzed for their expression of NKG2A or TIGIT against CD45 in tumors from CBL0137 treated and untreated (Vehicle) mice. Representative dot blots are presented.

**(B)** CD8^+^ T cells expressing GZB (red) were backgated on total CD8+ T cells (gray) and analyzed for their expression of NKG2A or TIGIT against CD45 in tumors from CBL0137 treated and untreated (Vehicle) mice. Representative dot blots are presented.

**(C)** CD4^+^ T_conv_ cells expressing NKG2A or TIGIT (red) were backgated on total CD4^+^ T_conv_ cells (gray) and analyzed for their expression of CD69 against CD45 in tumors from CBL0137 treated and untreated (Vehicle) mice. Representative dot blots are presented.
